# 24(S)-Saringosterol Prevents Cognitive Decline in a Mouse Model for Alzheimer’s Disease

**DOI:** 10.3390/md19040190

**Published:** 2021-03-27

**Authors:** Nikita Martens, Melissa Schepers, Na Zhan, Frank Leijten, Gardi Voortman, Assia Tiane, Ben Rombaut, Janne Poisquet, Nienke van de Sande, Anja Kerksiek, Folkert Kuipers, Johan W. Jonker, Hongbing Liu, Dieter Lütjohann, Tim Vanmierlo, Monique T. Mulder

**Affiliations:** 1 Department of Internal Medicine, Section Pharmacology and Vascular Medicine, Erasmus University Medical Center, 3015 Rotterdam, The Netherlands; n.martens@erasmusmc.nl (N.M.); n.zhan@erasmusmc.nl (N.Z.); f.leijten@erasmusmc.nl (F.L.); g.voortman@erasmusmc.nl (G.V.); mm.vandesande@student.maastrichtuniversity.nl (N.v.d.S.); tim.vanmierlo@uhasselt.be (T.V.); 2 Department of Neuroscience, Biomedical Research Institute, European Graduate School of Neuroscience, Hasselt University, BE 3590 Hasselt, Belgium; melissa.schepers@uhasselt.be (M.S.); assia.tiane@uhasselt.be (A.T.); ben.rombaut@uhasselt.be (B.R.); janne_poisquet@hotmail.com (J.P.); 3 Department of Psychiatry and Neuropsychology, School for Mental Health and Neurosciences, Division Translational Neuroscience, Maastricht University, 6200 Maastricht, The Netherlands; 4 Key Laboratory of Marine Drugs, Chinese Ministry of Education, School of Medicine and Pharmacy, Ocean University of China, Qingdao 266100, China; liuhongb@ouc.edu.cn; 5 Institute of Clinical Chemistry and Clinical Pharmacology, University Hospital Bonn, 53105 Bonn, Germany; Anja.Kerksiek@ukbonn.de (A.K.); Dieter.Luetjohann@ukbonn.de (D.L.); 6 Department of Pediatrics, Section of Molecular Metabolism and Nutrition, University Medical Center Groningen, 9713 Groningen, The Netherlands; f.kuipers@umcg.nl (F.K.); j.w.jonker@umcg.nl (J.W.J.)

**Keywords:** Alzheimer’s disease, seaweed, *Sargassum fusiforme*, phytosterols, cholesterol metabolism

## Abstract

We recently found that dietary supplementation with the seaweed *Sargassum fusiforme*, containing the preferential LXRβ-agonist 24(S)-saringosterol, prevented memory decline and reduced amyloid-β (Aβ) deposition in an Alzheimer’s disease (AD) mouse model without inducing hepatic steatosis. Here, we examined the effects of 24(S)-saringosterol as a food additive on cognition and neuropathology in AD mice. Six-month-old male APPswePS1ΔE9 mice and wildtype C57BL/6J littermates received 24(S)-saringosterol (0.5 mg/25 g body weight/day) (APPswePS1ΔE9 *n* = 20; C57BL/6J *n* = 19) or vehicle (APPswePS1ΔE9 *n* = 17; C57BL/6J *n* = 19) for 10 weeks. Cognition was assessed using object recognition and object location tasks. Sterols were analyzed by gas chromatography/mass spectrometry, Aβ and inflammatory markers by immunohistochemistry, and gene expression by quantitative real-time PCR. Hepatic lipids were quantified after Oil-Red-O staining. Administration of 24(S)-saringosterol prevented cognitive decline in APPswePS1ΔE9 mice without affecting the Aβ plaque load. Moreover, 24(S)-saringosterol prevented the increase in the inflammatory marker Iba1 in the cortex of APPswePS1ΔE9 mice (*p* < 0.001). Furthermore, 24(S)-saringosterol did not affect the expression of lipid metabolism-related LXR-response genes in the hippocampus nor the hepatic neutral lipid content. Thus, administration of 24(S)-saringosterol prevented cognitive decline in APPswePS1ΔE9 mice independent of effects on Aβ load and without adverse effects on liver fat content. The anti-inflammatory effects of 24(S)-saringosterol may contribute to the prevention of cognitive decline.

## 1. Introduction

Alzheimer’s disease (AD) is the most prevalent form of dementia in the elderly. This neurodegenerative disorder is characterized by a progressive cognitive decline, accumulation of amyloid-β (Aβ), formation of neurofibrillary tangles, neuroinflammation, and loss of neurons and synapses [1]. Although symptomatic treatment can help to temporarily reduce symptoms, currently available treatments cannot effectively prevent AD pathology [2]. Accumulating evidence points to a role for an imbalanced cholesterol turnover in the central nervous system in AD pathology [3,4,5,6,7,8]. In line with this, a major genetic risk factor for sporadic AD is the ε4 isoform of the *APOE* gene. APOE encodes the cholesterol carrier apolipoprotein (Apo) E, the main extracellular lipid transporter in the central nervous system [4,9]. Moreover, chronic neuroinflammation attributed to sustained or excessive activation of microglia has been demonstrated to exacerbate AD pathologies [10].

Liver X receptors (LXRα and -β) are oxysterol-activated nuclear receptors that not only act as master regulators of cellular cholesterol and lipid metabolism but also control inflammatory processes [11,12], making these nuclear receptors interesting targets in AD. The use of synthetic pan LXRα/β agonists (e.g., T0901713 and GW3965) has been shown to prevent cognitive decline in various animal models of AD [12,13,14,15,16]. However, the findings on the impact of LXR activation on Aβ plaque deposition and neuroinflammation remain inconclusive [3,13,14,15,17]. Yet, adverse effects of these LXR agonists, including hypertriglyceridemia and hepatic steatosis, impede their transfer to clinical application [18,19,20]. Interestingly, hepatic LXRα activation is known to promote hepatic lipogenesis and is considered responsible for the adverse effects observed upon pan LXRα/β activation [20,21]. Compounds preferentially activating LXRβ may therefore be superior therapeutic agents for the prevention of AD.

By virtue of their distinct nutritional composition, various health benefits have been attributed to seaweeds [22,23]. They are a source of a variety of bioactive compounds and secondary metabolites, some of which exert neuroprotective effects such as Aβ-lowering, anti-inflammatory and anti-oxidative effects that can help in managing neurodegenerative diseases such as AD [24]. Compounds present in seaweed reported to have these neuromodulatory effects include oligomannate [25], fucoidan [26], *Sargassum fusiforme* polysaccharide 65 [27], fucosterol [28,29,30], fucoxanthin [28,31,32,33,34,35], and 24(S)-saringosterol [21]. We previously reported that the seaweed *Sargassum fusiforme*, as well as purified 24(S)-saringosterol, preferentially activate LXRβ [21] and therefore are thought not to induce hepatic lipid accumulation [36]. We demonstrated that the saringosterol-containing brown seaweed *Sargassum fusiforme*, as well as its lipid extracts, can prevent cognitive impairment and reduce Aβ plaque load in an AD mouse model without causing the adverse effects induced by pan synthetic LXR agonists [21]. Moreover, we demonstrated that 24(S)-saringosterol reduced neuronal Aβ secretion in vitro while promoting microglia-mediated clearance of Aβ [21]. Here we examined the contribution of purified 24(S)-saringosterol to the beneficial effects of *Sargassum fusiforme* on cognition and neuropathology in APPswePS1ΔE9 mice. We show that the phytosterol 24(S)-saringosterol prevented cognitive decline in APPswePS1ΔE9 mice, despite having no effect on Aβ plaque load. Our data point to the immunomodulatory effects of 24(S)-saringosterol treatment contributing to the observed neuroprotective properties.

## 2. Results

### 2.1. 24(S)-Saringosterol Prevents Cognitive Decline in APPswePS1ΔE9 Mice

**Figure 1:**
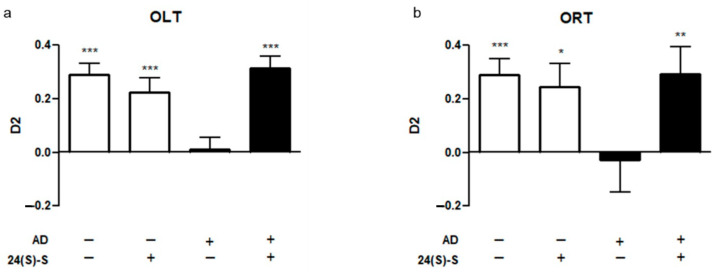
24(S)-Saringosterol prevents cognitive decline in APPswePS1∆E9 mice. The impact of a 10-week 24(S)-saringosterol or vehicle administration on cognitive functioning in WT and APPswePS1ΔE9 (AD) mice was determined using an object location task (OLT; 4 h inter-trial interval (ITI)) (**a**) and an object recognition task (ORT; 1 h ITI) (**b**). Bars represent mean ± SEM (OLT: *n* = 18, 19, 16, and 18; ORT: *n* = 13, 14, 15, and 17 per group, respectively)). D2 values relative to 0: * *p* ≤ 0.05, ** *p* ≤ 0.01, *** *p* ≤ 0.001.

To test the effect of 10-week administration of 24(S)-saringosterol on cognition of WT and APPswePS1ΔE9 mice, the mice were subjected to an object location task (OLT) and an object recognition task (ORT). Cognitive decline in APPswePS1ΔE9 mice was significantly prevented by daily administration of 24(S)-saringosterol, as shown by enhanced spatial and object memory as assessed with OLT and ORT (Figure 1a,b). Vehicle-treated APPswePS1ΔE9 mice could not discriminate the displaced or novel object in their environment (*p* = 0.787 and *p* = 0.807 for OLT and ORT, respectively), while memory was intact in APPswePS1ΔE9 mice that received 24(S)-saringosterol (*p* < 0.01 and *p* < 0.01) as well as in WT mice that received the vehicle control (*p* < 0.001 and *p* < 0.001) or 24(S)-saringosterol (*p* < 0.01 and *p* < 0.05) (Figure 1a,b).

### 2.2. 24(S)-Saringosterol Detectable in the Circulation and the Brain after Administration

**Figure 2:**
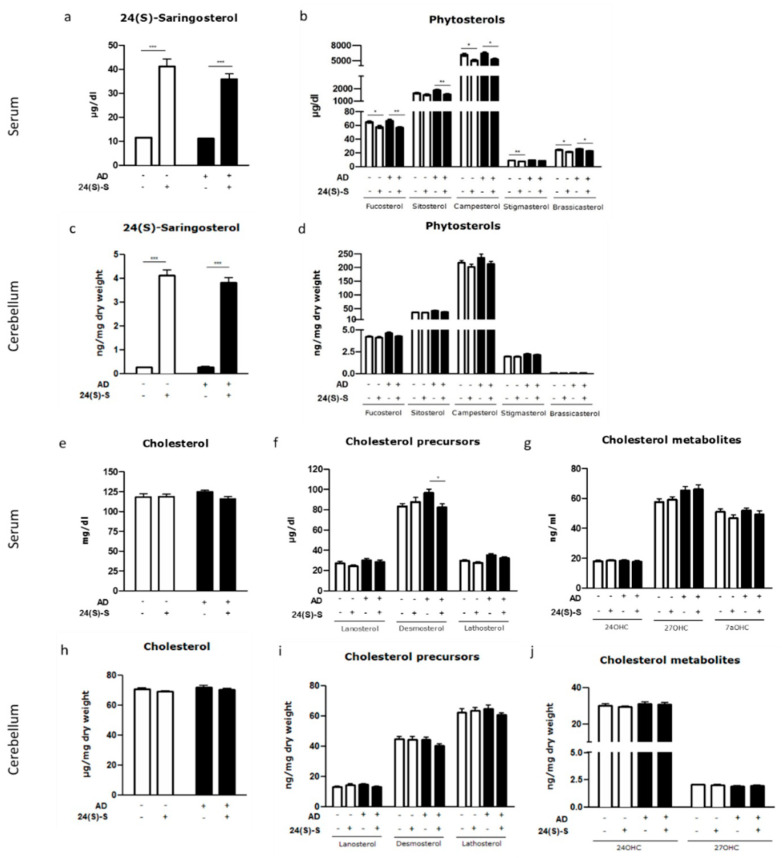
Sterol concentrations in serum and cerebellum after 10-week 24(S)-saringosterol or vehicle administration. Concentrations of 24(S)-saringosterol (**a**,**c**), phytosterols (**b**,**d**), cholesterol (**e**,**h**), cholesterol precursors (**f**,**i**), and metabolites (**g**,**j**) in serum and cerebellum samples of WT and APPswePS1ΔE9 (AD) mice receiving either vehicle or 24(S)-saringosterol. Bars represent mean ± SEM (*n* ≥ 16, 13, 15, and 18 per group, respectively). * *p* ≤ 0.05, ** *p* ≤ 0.01, *** *p* ≤ 0.001.

Administration of 24(S)-saringosterol increased its concentrations in serum from 11.4 ± 0.5 to 38.5 ± 12.0 µg/dL (F(1, 69) = 176.418, *p* < 0.001) and in the cerebellum from 0.3 ± 0.04 to 4.0 ± 0.9 ng/mg dry weight (F(1, 69) = 576.110, *p* < 0.001) (Figure 2a,c). The 24(S)-saringosterol detected in mice that were fed chow is likely derived from avenasterol that is present in terrestrial plants.

Furthermore, 24(S)-saringosterol treatment resulted in decreased concentrations of fucosterol in the circulation (F(1, 70) = 19.535, *p* < 0.001) and in the cerebellum (F(1, 68) = 4.851, *p* < 0.05) (Figure 2b,d). Serum concentrations of sitosterol (F(1, 70) = 11.072, *p* = 0.001), campesterol (F(1, 68) = 19.236, *p* < 0.001), stigmasterol (F(1, 68) = 17.951, *p* < 0.001), and brassicasterol (F(1, 66) = 15.026, *p* < 0.001) also decreased (Figure 2b). With exception of fucosterol, phytosterol concentrations in the cerebellum remained unaffected (Figure 2d). The concentration of cholesterol, its precursors and metabolites in serum and in the cerebellum remained unaffected by 24(S)-saringosterol administration, except for a decrease in serum desmosterol concentrations in APPswePS1ΔE9 mice (*p* = 0.050), but not in WT mice (Figure 2e–j).

### 2.3. 24(S)-Saringosterol Does Not Affect the Aβ Plaque Load in the Cortex and the Hippocampus

**Figure 3:**
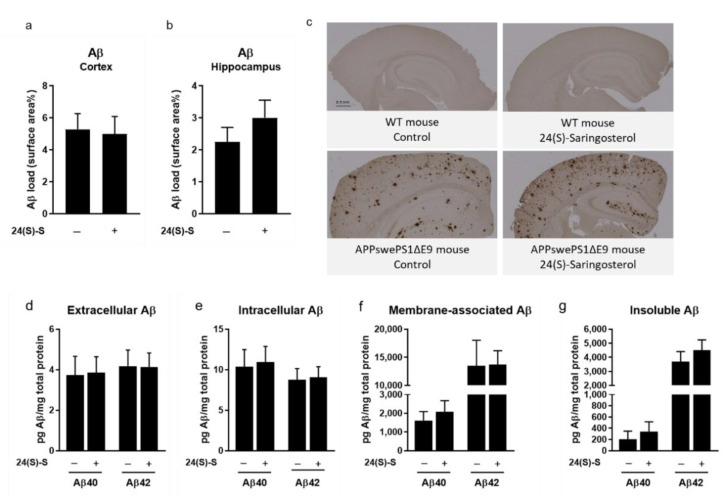
No effect of 24(S)-saringosterol administration on Aβ plaque load in APPswePS1∆E9 mice. The percentage of surface coverage of the Aβ staining was determined in the total cortical (**a**) and hippocampal area (**b**) of APPswePS1ΔE9 mice after immunohistochemical staining (cortex: *n* = 6 per group; hippocampus: *n* = 5 and 6 per group, respectively). Photos of Aβ-stained cortical and hippocampal areas representative for the experimental groups are shown (**c**). Soluble (extracellular (**d**), intracellular (**e**), and membrane-associated (**f**)) Aβ and insoluble Aβ (**g**) in the cortex of APPswePS1ΔE9 mice administered with vehicle or 24(S)-saringosterol was quantified using ELISA (*n* = 16 and 20 per group, respectively). Bars represent mean ± SEM.

The Aβ plaque load in the cortex (*p* = 0.963) and in the hippocampus (*p* = 0.450) of APPswePS1ΔE9 mice (Figure 3a,b) were not affected by 24(S)-saringosterol administration. There were also no differences in concentrations of insoluble Aβ_40_ and Aβ_42_ or soluble extracellular, intracellular, or membrane-associated Aβ_40_ and Aβ_42_ (*p* > 0.05) (Figure 3d–g).

### 2.4. 24(S)-Saringosterol Prevents the Increase in the Expression of Microglia Activation Marker Iba1 in APPswePS1ΔE9 Mice

**Figure 4:**
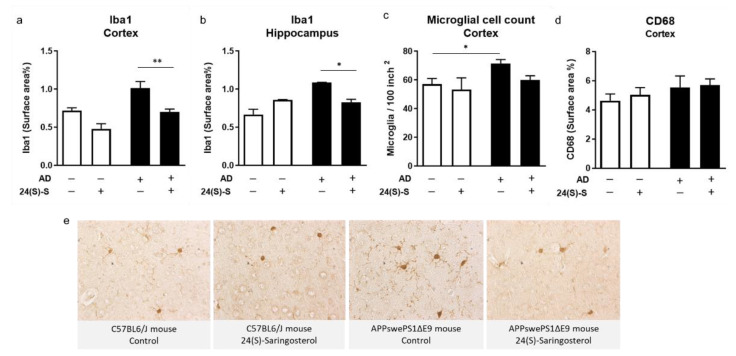
Administration of 24(S)-saringosterol reduces the microglia marker Iba1 and microglial density, but not CD68 in APPswePS1ΔE9 mice. Coronal sections of the brain of WT and APPswePS1ΔE9 (AD) mice were stained for Iba1 (**a**–**c**,**e**) and CD68 (**d**) by immunohistochemistry, and the percentage of surface coverage of the staining in the total cortical and hippocampal area was determined (**a**,**b**,**d**). Photos of the Iba1-stained cortex representative for the experimental groups are shown (e). Iba1 and CD68 levels are presented as the percentage of surface coverage, the microglia cell count as the number of Iba1-positive stained cell bodies per 100 inch^2^ cortex. Bars represent mean ± SEM (*n* = 5–6, 3, 3–6, and 5 per group, respectively). * *p* ≤ 0.05, ** *p* ≤ 0.01.

To test the potential involvement of microglia in the neuromodulatory effects of 24(S)-saringosterol, we investigated the impact of 24(S)-saringosterol administration on the microglia/macrophage markers ionized calcium-binding adapter molecule 1 (Iba1) and cluster of differentiation 68 (CD68). Compared to WT mice, Iba1 levels, as determined by the surface area%, were higher in the cortex and hippocampus of APPswePS1ΔE9 mice (F(1,15) = 17.777, *p* = 0.001 and F(1,12) = 12.626, *p* < 0.01, respectively), and decreased upon 24(S)-saringosterol administration (*p* < 0.01 and *p* < 0.05, respectively) (Figure 4a,b). In line with this observation, the microglia cell count in the cortex was higher in APPswePS1ΔE9 mice than in WT mice (F(1, 15) = 7.871, *p* < 0.05) (Figure 4c). The difference in microglia cell count in the cortex of APPswePS1ΔE9 mice and WT mice on the vehicle treatment (*p* < 0.05) disappeared upon 24(S)-saringosterol administration (*p* = 0.718). There were no differences in CD68 levels in the cortex of WT and APPswePS1ΔE9 mice (F(1, 17) = 0.969, *p* = 0.339), and no effects of 24(S)-saringosterol treatment (F(1, 17) = 0.109, *p* = 0.746) (Figure 4d).

### 2.5. 24(S)-Saringosterol Affects the Expression of LXR Target Genes In Vitro, But Not In Vivo

**Figure 5:**
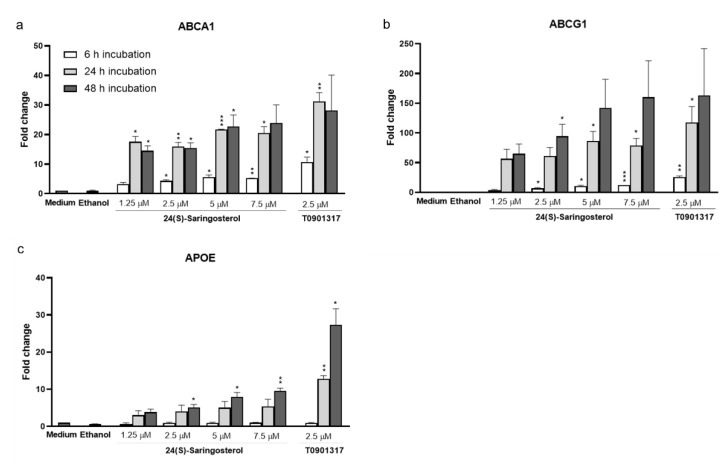
Effect of 24(S)-saringosterol on the expression of LXR target genes in CCF-STTG1 cells. Gene expression of *ABCA1* (**a**), *ABCG1* (**b**), and *APOE* (**c**) was determined in CCF-STTG1 cells incubated with 24(S)-saringosterol (1.25–7.5 µM) for 6 h, 24 h, and 48 h or a control (DMEM/F-12 medium (48 h), ethanol (48 h) or the synthetic pan LXRα/β agonists T0901317 (2.5 µM, 6 h)). Gene expression was normalized to the most stable housekeeping gene (*SDHA)* and expressed as fold change compared to the medium control. The fold change values are the means of three experiments ± SEM (*n* = 3). Compared to the control (DMEM/F-12 medium) value: * *p* ≤ 0.05, ** *p* ≤ 0.01, *** *p* ≤ 0.001.

We assessed the effect of 24(S)-saringosterol on the expression of lipid metabolism-related LXR target genes in CCF-STTG1 cells (Figure 5) and in the hippocampus of APPswePS1ΔE9 mice (Supplementary Figure S1). Incubation of CCF-STTG1 glial cells with 24(S)-saringosterol increased the expression of *ABCA1* (Figure 5a), *ABCG1* (Figure 5b), and *APOE* (Figure 5c) in a dose-dependent manner. The expression of *ABCA1* and *ABCG1* was increased to a comparable extend by the positive control T0901317, while the expression of *APOE* was increased to a lesser extent by 24(S)-saringosterol than by T0901317. However, no effect of 24(S)-saringosterol administration could be detected on the expression of *Abca1*, *Abcg1*, *Apoe*, *Scd1*, or *Srebf1* in the hippocampus of WT or APPswePS1ΔE9 mice (*p* > 0.05) (Supplementary Figure S1).

### 2.6. 24(S)-Saringosterol Administration Does Not Induce Accumulation of Triglycerides in the Liver or in Serum

**Figure 6:**
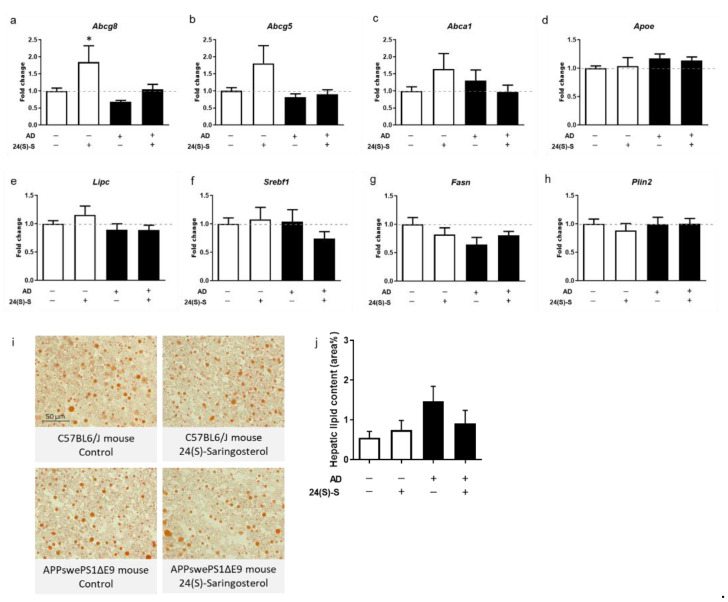
No effect of 24(S)-saringosterol administration on hepatic neutral lipid accumulation in the liver of WT or APPswePS1ΔE9 mice. Gene expression of *Abcg8* (**a**), *Abcg5* (**b**), *Abca1* (**c**), *Apoe* (**d**), *Lipc* (**e**), *Srebf1*(**f**), *Fasn* (**g**), and *Plin2* (**h**) was analyzed in livers of WT and APPswePS1ΔE9 mice treated with 24(S)-saringosterol or vehicle (*n* ≥ 8 per group). Neutral lipids in livers of WT and APPswePS1ΔE9 (AD) mice were stained with Oil Red O. Photos of representative IHC staining are shown (**i**). The surface area of the Oil-Red-O-stained lipids in the livers was quantified and is presented as the percentage of surface coverage (**j**) (*n* ≥ 13 per group). The bars represent mean ± SEM. * *p* ≤ 0.05.

Treatment with 24(S)-saringosterol increased the expression of *Abcg8* in the liver (F(1, 37) = 8.533, *p* < 0.01) (Figure 6a), while the expression of *Abcg5*—which was higher in WT mice than in APPswePS1ΔE9 mice (F(1, 34) = 4.234, *p* < 0.05)—, *Abca1*, and *Apoe* was not significantly increased (Figure 6b–d). The expression of *Lipc, Srebf1, Fasn*, and *Plin2* remained unaffected (Figure 6e–h). Administration of 24(S)-saringosterol did not affect the neutral lipid content in the liver of WT or APPswePS1ΔE9 mice (Figure 6i,j). Because of the observed high variability in the neutral lipid content in the livers of all 4 groups, an additional group of WT mice was administered vehicle or saringosterol for 3 weeks.

Three weeks of saringosterol administration did not induce accumulation of neutral lipids in the liver of WT mice (Supplementary Figure S2a). Neither did it affect triglyceride or cholesterol concentrations in serum (*p* = 0.683 and *p* = 0.562, respectively) (Supplementary Figure S2b,c). The expression levels of *Lipc*, *Srebf1*, *Abca1*, *Abcg1*, *Apoe*, and *Lxra* in the liver of these mice were not affected by 24(S)-saringosterol administration, although the expression of *Fasn* did increase (t(8) = −2.64, *p* < 0.05) (Supplementary Figure S2d–j).

## 3. Discussion

In this study, we examined whether purified 24(S)-saringosterol can preserve cognition and prevent the development of neuropathology in a mouse model for AD. Our data show that 10 weeks of administration with the semi-synthetic preferential LXRβ activating phytosterol 24(S)-saringosterol prevented cognitive decline in APPswePS1ΔE9 mice, without affecting the Aβ plaque load. Concentrations of 24(S)-saringosterol in the brain were significantly increased upon 24(S)-saringosterol administration, and microglial activation in the brain of APPswePS1ΔE9 mice was found to be reduced.

The prevention of the cognitive decline in APPswePS1ΔE9 mice upon 24(S)-saringosterol administration is in accordance with our previously reported data demonstrating neuroprotective effects of T0901317 and 24(S)-saringosterol-containing *Sargassum fusiforme* or its lipid extract [14,21]. Although 24(S)-saringosterol reduces neuronal Aβ_42_ release and promotes microglial Aβ clearance *in vitro* [21], no effect of 24(S)-saringosterol administration on Aβ plaque load could be detected. This observation is in line with our data showing no effect of T0901317 on Aβ plaque load despite effects on cognition [14]. However, the mice treated with T0901713 were much older than the mice in the present study and therefore Aβ deposition could not be prevented. Because *Sargassum fusiforme*, either in crude form or as a lipid extract, did decrease the Aβ plaque load in APPswePS1ΔE9 mice [21], constituents other than saringosterol are likely to reduce the deposition of Aβ. Aβ plaque lowering effects have been reported for several constituents besides 24(S)-saringosterol contained by *Sargassum fusiforme* including the phytosterols β-sitosterol [37,38] and stigmasterol [39,40], the carotenoid fucoxanthin [28,31,32], and oligosaccharide sodium oligomannate [24]. These findings suggest that LXR activation by 24(S)-saringosterol can prevent the decline in cognition independently of its effect on Aβ deposition.

Although 24(S)-saringosterol concentrations were significantly increased in serum and in the brain of the mice after its administration, this did not affect the concentrations of cholesterol, its precursors, or metabolites. Phytosterol concentrations in serum were reduced, possibly as a result of competition for incorporation in micelles and the subsequent intestinal absorption or an inhibitory effect of 24(S)-saringosterol on intestinal sterol absorption. An alternative explanation is an enhancing effect of 24(S)-saringosterol on sterol excretion via LXR activation and upregulation of Abcg5/8 in the liver and the intestine [41,42]. The expression of lipid metabolism-related LXR target genes in the brain remained unaffected despite the increased concentrations of 24(S)-saringosterol. However, in cultured CCF-STTG1 cells, we observed that 24(S)-saringosterol administration did increase the expression of LXR target genes *ABCA1*, *ABCG1*, and *APOE*. Chen et al. (2014) obtained similar results after saringosterol administration to HEK293T, HepG2, THP-1 monocytes, and RAW264.7 cells [36]. The absence of an effect *in vivo* may be caused by the chronic nature of the 24(S)-saringosterol administration leading to a new balance in gene expression levels or the difference in 24(S)-saringosterol concentration *in vivo* (9 × 10^−4^ mM in serum) compared to *in vitro* (1.25–7.5 mM). Previously, we observed a limited increase in expression of LXR target genes in the brains of WT and APPswePS1ΔE9 mice upon administration of *Sargassum fusiforme* containing a similar concentration of 24(S)-saringosterol [21]. *Sargassum fusiforme* contains additional LXR agonists, including fucosterol, that may contribute to the gene expression profile resulting from *Sargassum fusiforme* supplementation. Therefore, it remains to be established via which biological pathways, involving LXR activation or not, 24(S)-saringosterol exerts its neuroprotective effects in AD mice.

Activation of LXR is known to transrepress inflammatory pathways in immune cells in the central nervous system through SUMOylation [43]. Because chronic neuroinflammation—attributed to excessive activation of microglia—exacerbates AD pathologies [10], activating LXR might alleviate AD symptoms by dampening this process. This is supported by our findings that 24(S)-saringosterol administration did restore the expression of Iba1 (a marker for microglial activation and inflammation [44]) and the number of microglia in the AD mice to levels similar to those in WT mice. On the other hand, the absence of differences in the expression of CD68 suggests no involvement of phagocytic activity of microglia [45]. Increased Iba1 expression, and thus microglial activation, in brain tissue of AD patients has been reported [46]. An increased ApoE production may contribute to the reduction in neuroinflammation [21,47]. However, an upregulated ApoE production was induced by 24(S)-saringosterol only *in vitro* and not *in vivo* [21]. Therefore, the exact immunomodulatory effects of 24(S)-saringosterol in AD remain to be elucidated.

Our observation that administration of 24(S)-saringosterol as a preferential LXRβ activator does not induce hepatic steatosis is in line with the assumption that triglyceride accumulation in the liver is predominantly driven by LXRα activation [19]. Further research should elaborate on the potential adverse effects of long-term 24(S)-saringosterol administration. Our data therefore support the potential application of pure 24(S)-saringosterol, *Sargassum fusiforme*, either crude or as an extract, in the prevention and retardation of AD-related symptoms.

An advantage to the use of crude seaweed or a seaweed extract over pure 24(S)-saringosterol could be the presence of constituents with potential additional or synergistic effects. Seaweed constituents other than 24(S)-saringosterol have been reported to display beneficial effects in AD models [24]. Several phytosterols, including fucosterol, sitosterol, stigmasterol, and brassicasterol, have been reported to exert anti-inflammatory effects via LXR activation [24,48,49]. However, we could not confirm these results in an LXR assay using physiological sterol concentrations [21]. Evidence indicates that sitosterol can augment the polarization of macrophages towards an anti-inflammatory phenotype via transrepression of toll-like receptor activation [48]. Moreover, the formation of Aβ can be reduced by sitosterol [38], stigmasterol [39,40], and fucosterol [28]. Fucosterol also alleviates Aβ-induced ER stress in primary rat hippocampal neurons and prevents soluble Aβ_42_ exposure-induced cognitive decline in aging rats [29]. The carotenoid fucoxanthin may contribute to the prevention of AD-related symptoms by its anti-oxidative properties [34] and by preventing the formation of Aβ peptides [28,31,32,33] or Aβ neurotoxicity [31]. Furthermore, seaweed-derived polyphenols such as phloroglucinol have been reported to prevent cognitive impairment in AD mice, possibly via anti-oxidative effects [49], and fucoidans were found to ameliorate cognitive impairments in models for neurodegeneration via anti-oxidative or anti-inflammatory mechanisms [26]. The oligosaccharide sodium oli-gomannate, which has demonstrated cognitive improvement in a phase 3 clinical trial in China, was found to reduce neuroinflammation by remodeling the gut microbiome [25]. Furthermore, seaweeds may have a beneficial effect on cognition by being a low glycemic food, tending to release glucose slowly and steadily [50]. Because a steady supply of glucose to the brain is required for optimal cognitive performance and an impaired glucose metabolism has been linked to AD, blood glucose control is crucial [50,51,52]. Therefore, dietary supplementation with seaweed combines the beneficial properties of multiple constituents that may act synergistically in the prevention and retardation of AD-related symptoms.

In conclusion, we showed that semi-synthetic purified 24(S)-saringosterol, which is bioavailable in the central nervous system, prevents cognitive decline in a well-established AD mouse model, despite having no effect on Aβ plaque load, or any detectable effects on lipid or cholesterol homeostasis. Our data point to immunomodulatory effects of 24(S)-saringosterol contributing to its neuroprotective properties, but detailed mechanisms remain to be elucidated. Moreover, 24(S)-saringosterol can be regarded as a promising agent for the prevention of deterioration of AD-related symptoms.

## 4. Materials and Methods

### 4.1. Route of 24(S)-Saringosterol Administration

Prior to this experiment, two routes of 24(S)-saringosterol administration were compared: oral gavage and subcutaneous injection. Six C57BL6/J mice received an oral gavage and six C57BL6/J mice received a subcutaneous injection containing 0.5 mg 24(S)-saringosterol—semi-synthesized from kelp-derived fucosterol—(purity of 98.2%; COMFiON B.V., Leimuiden, The Netherlands) per 25 g body weight twice-daily for three consecutive days, whereafter sterol concentrations in serum, brain, and other tissues were determined by gas chromatography/mass spectrometry (GC/MS) as described previously [21,53] (Supplementary Table S1). Oral gavage and subcutaneous injection led to saringosterol concentrations of 40.9 ± 12.7 and 28.0 ± 3.3 µg/dL in serum and to 6.43 ± 1.40 and 5.51 ± 1.85 ng saringosterol per mg dry weight cerebellum. Because the bioavailability of saringosterol was higher upon oral gavage, we administered saringosterol via this route.

### 4.2. Animals and Diet

Male APPswePS1ΔE9 (AD) and wildtype C57BL6/J (WT) littermate mice were obtained by backcrossing male APPswePS1ΔE9 mice (The Jackson Laboratory, Bar Harbor, ME, USA) with female C57BL6/J mice (Envigo, Horst, The Netherlands). The animals were housed in a conventional animal facility at Hasselt University. Two weeks prior to the start of the behavioral experiments, 5-month old mice were housed individually. Mice were fed ad libitum and kept in an inversed 12/12 h light/dark cycle with behavioral experiments performed during the dark phase of the cycle. The cognitive performances were scored blindly. The body weight of the mice was monitored twice a week. Two series of animal experiments were conducted. The animal procedures were approved by the ethical committee for the animal experiments of Hasselt University and performed in accordance with institutional guidelines (protocol ID201849). From 6 months of age, mice received a daily oral gavage containing 0.5 mg 24(S)-saringosterol (COMFiON B.V.; APPswePS1ΔE9: *n* = 20, C57BL6/J littermates: *n* = 19) in 200 μL of vehicle (0.5% methylcellulose containing 1% ethanol and 2% Tween) per 25 g body weight or vehicle only (APPswePS1ΔE9: *n* = 17; C57BL6/J: *n* = 19) for 10 consecutive weeks. The body weight of the mice (41.4 ± 6.0 g) was stable over the treatment period with no effect of diet (F(1, 35) = 0.689, *p* = 0.412) or genotype (F(1, 35) = 2.373, *p* = 0.132). To test for potential adverse effects of saringosterol administration on lipid metabolism, a second batch of C57BL6/J mice (Envigo RMS BV, Venray, The Netherlands) received a daily oral gavage containing 0.5 mg saringosterol (purity of 100%; a ratio of 24(S)/24(R)-saringosterol of 1:1)—semi-synthesized from commercially available hyodeoxycholic acid—in 200 μL vehicle (0.5% methylcellulose containing 1% ethanol and 2% Tween) per 25 g body weight (*n* = 7) or vehicle only (*n* = 7) for 3 weeks from the age of 12 weeks.

### 4.3. Cognitive Testing

Prior to the baseline assessment, the mice were habituated to the arena and to the four different objects used for cognitive testing, as previously described [21]. At baseline, a functional memory of both WT and APPswePS1ΔE9 mice was confirmed with the OLT. After one resting day, the experiment was initiated. The ORT and the OLT were conducted after the treatment period of 10 weeks by a researcher that was blinded to the experimental groups. The same objects were used for the ORT and OLT and objects were selected following a randomized scheme (Supplementary Tables S2 and S3).

The ORT was conducted as described previously [54]. During the first trial (T1), the animal was exposed to two similar objects for 4 min after which it was placed back in its home cage. After a 1 h inter-trial interval (ITI), a second trial (T2) was performed during which the animal was exposed for 4 min to one familiar object from T1 and one novel object. The times spent exploring each object during T1 and T2 were recorded manually. Biting or sitting on the object was not considered exploratory behavior. As a measure of object memory, the discrimination index (D2) ((exploration time for novel object)—(exploration time for familiar object)/(total exploration time in T2)) in T2 was calculated.

The OLT was conducted as a modified form of the ORT described elsewhere [54]. During the first trial (T1), the animal was exposed to two similar objects placed symmetrically in the arena center for 4 min, after which it was placed back in its home cage. After a delay interval of 4 h, a second trial (T2) was performed during which the animal was exposed for 4 min to the two objects from T1 of which one was displaced. The times the mice spent exploring each object during T1 and T2 were recorded manually. As a measure of spatial memory, the discrimination index (D2) ((exploration time for displaced object) —(exploration time for stationary object)/(total exploration time in T2)) in T2 was calculated.

### 4.4. Tissue Sample Preparation

After the post-treatment memory assessment, mice were sacrificed and tissues were isolated for further analyses. Mice were anesthetized by intraperitoneal injection of Dolethal (Vetoquinol, Aartselaar, Belgium) (200 mg per kg body weight) followed by transcardial perfusion with Heparin-phosphate-buffered saline (PBS). Blood was collected via cardiac puncture and was centrifuged for 10 min at 200 g to separate serum, which was stored at −80 °C until use. Brains were isolated and divided into the forebrain (above bregma 0), the cerebellum, and the remaining two hemispheres. The cerebellum was snap-frozen and stored at −80 °C until sterol profiling. The left hemisphere was fixed in formalin and embedded in paraffin for immunohistochemistry. The cortex from the right hemisphere was snap-frozen and cryopreserved for Aβ ELISA analyses. Half of the liver was directly snap-frozen for mRNA expression analyses, the other half was stored at −80 °C in O.C.T. embedding compound (Sakura Finetek USA, Inc., Torrance, CA, USA) for Oil Red O staining.

### 4.5. Determination of Lipid Profile

Sterol profiles in serum and in the cerebellum were determined by GC/MS as described previously [21,53]. In short, prior to sterol analysis, the brain tissue samples (cerebellum) were spun in a speed vacuum dryer to relate individual sterol concentrations to dry weight. The sterols were extracted from the dried tissues by placing them in a 5-mL mixture of chloroform-methanol. Subsequently, 1 mL of the brain sterol extracts was evaporated to dryness. Furthermore, 1 mL of distilled water was added to the samples. To extract the neutral sterols, 3 mL of cyclohexane was added twice. The combined cyclohexane phases were again evaporated to dryness under a stream of nitrogen at 63 °C, and the sterols were dissolved in 100 μL n-decane. After transfer to gas-chromatography (GC)-vials, the sterols were converted to trimethylsilyl ethers (TMSis) and incubated at 60 °C for 1 h [7]. Levels of cholesterol were determined in a gas-chromatograph-flame ionization detector (GC-FID) with 50 μL 5α-cholestane-solution (1 mg/mL 5α-cholestane in cyclohexane) as an internal standard. Levels of plant sterols (campesterol, sitosterol), cholesterol precursors (lanosterol, lathosterol, and desmosterol), and cholesterol metabolites (24S-OH-cholesterol and cholestanol) were determined using gas chromatography-mass spectrometry (GC-MS) using epicoprostanol as an internal standard.

Triglyceride and cholesterol concentrations in serum were determined with enzymatic reagent kits according to the manufacturer’s instructions (DiaSys Diagnostic Systems, Holzheim, Germany).

### 4.6. Immunohistochemistry—Quantification of Aβ, Iba1 and, CD68

For quantification of Aβ, Iba1 (also known as allograft inflammatory factor 1 (AIF-1)), and CD68 using immunohistochemistry, the left hemispheres of the brains were fixed in 10% formalin and embedded in paraffin by incubation in increasing concentrations of ethanol (70% (1 h), 80% (1 h), 95% (1 h), 100% (1.5 h)), xylene (1.5 h), and paraffin wax (2 h at 60 °C) using a Thermo Scientific Excelsior ES Tissue Processor (Thermo Fisher Scientific, Waltham, MA, USA). Embedded hemispheres were cut with an HM 340E Electronic Rotary Microtome (Thermo Fisher Scientific, Waltham, MA, USA) to obtain 4 µm sections which were mounted on glass slides, air-dried overnight, and stored at room temperature until use. For Aβ, Iba1, and CD68 staining, sections were deparaffinized (incubation in xylene (10 min) and decreasing concentrations of ethanol (100% (6 min), 96% (3 min), 70% (3 min))), rinsed with TBS/0.3% Triton X-100 and incubated in 10 mM citrate buffer (pH 6.0) for 10 min at 100 °C. After blocking endogenous peroxidases by incubating with 3% H_2_0_2_/methanol for 10 min, sections were rinsed with TBS/0.3% Triton X-100 and incubated with blocking solution (5 v/v% bovine serum albumin in 1xTBS) for 1 h. Thereafter, sections were incubated overnight with the primary antibody in blocking solution (1:8000 Aβ antibody (clone 3D6), 1:1000 Iba1 antibody (Wako Chemicals USA, Inc., Richmond, VA: 019-19741), or 1:235 CD68 antibody (Santa Cruz Biotechnology Inc.: sc-9139, Dallas, TX, USA)) at 4 °C. After rinsing with TBS/0.3% Triton X-100, sections were incubated with appropriate biotinylated secondary antibodies for 30 min at room temperature followed by 30 min of incubation with avidin-biotin-complex (ABC kit, Vector Laboratories, Burlingame, CA, USA) and 5 min of incubation with diaminobenzidine (ImmPACT DAB, Vector). Sections were counterstained with hematoxylin, dehydrated (1 min incubation in 70% ethanol, 100% ethanol, and xylene), and covered with a coverslip. Digital images of the sections were obtained using a Leica DRMB microscope (Leica Microsystems, Rijswijk, The Netherlands) equipped with Leica Applications Suite software (Leica Microsystems, Rijswijk, The Netherlands). The surface area of the staining was quantified using Fiji ImageJ software by defining the pixel intensity of the staining in the total cortical or hippocampal area.

### 4.7. ELISA—Quantification of Aβ

For quantification of Aβ using ELISA, the cortex of the right hemisphere of the brains of APPswePS1ΔE9 mice was homogenized in TBS/0.1% Triton X-100 containing 2% complete protease inhibitor cocktail (Roche Diagnostics Ltd., Mannheim, Germany) (pH 7.2) and centrifuged (21,000× *g*, 10 min). The supernatant containing the extracellular soluble Aβ was obtained and stored at −80 °C until use, the pellet was sonicated in TBS containing 2% complete protease inhibitor cocktail (Roche Diagnostics Ltd.) and centrifuged (21,000× *g*, 10 min). The supernatant containing intracellular soluble Aβ was obtained and stored at −80 °C until use, the pellet was sonicated in 2% sodium dodecyl sulfate (SDS) in distilled water and centrifuged (21,000× *g*, 10 min). The supernatant containing membrane-associated soluble Aβ was obtained and stored at −80 °C until use, the pellet was sonicated in 70% formic acid in distilled water and centrifuged (44,000× *g*, 10 min). The supernatant containing insoluble Aβ was obtained and stored at −80 °C until use. In the obtained samples, Aβ_40_ and Aβ_42_ levels were quantified using an Aβ_40_ and Aβ_42_ ELISA (Invitrogen, Carlsbad, CA, USA) and related to the total protein content (in the extracellular soluble Aβ fraction) determined with a BCA protein assay kit (Thermo Fisher Scientific, Waltham, MA, USA) according to the manufacturer’s instructions.

### 4.8. Cell Culture—CCF-STTG1 Cells

Human Caucasian astrocytoma cells (CCF-STTG1) (Sigma-Aldrich, Saint Louis, MO, USA) cultured in DMEM/F-12 medium (Thermo Fisher Scientific) containing 10% heat-inactivated fetal calf serum (FCS) (Thermo Fisher Scientific, Waltham, MA, USA) and 1% 10,000 U penicillin/10,000 μg streptomycin/mL (Thermo Fisher Scientific, Waltham, MA, USA) at 37 °C and 5% CO_2_ were seeded in a 6-well plate with a density of 700,000 cells/well. The cells (passage 18–25) were incubated with 1.25, 2.5, 5.0, or 7.5 µM 24(S)-saringosterol or a negative or positive control (DMEM/F-12, ethanol or T0901317 (2.5 µM)) for 6, 24, or 48 h.

### 4.9. RNA Isolation and RT-Q-PCR

Hippocampus and liver tissue were homogenized using the BioSpec Mini-Beadbeater (Biospec Products, Bartlesville, OK, USA) and CCF-STTG1 cells were washed with cold PBS. RNA was prepared using Trizol (Invitrogen, Carlsbad, CA, USA) and RNA was reverse transcribed to cDNA using the QuantiTect Reverse Transcription Kit (Qiagen), according to the manufacturer’s instructions. Quantitative PCR (qPCR) was conducted, as previously described, on a CFX384 Thermal Cycler (Bio-Rad Laboratories) using SYBR Green PCR Select Master Mix (Applied Biosystems, Warrington, UK) [55]. Relative quantification of gene expression was accomplished by using the comparative Ct method. Data were normalized to the most stable reference genes (*Actb*, *B2m*, *Hprt1,* and *Sdha* (hippocampus) or *Actb* (liver)), which were analyzed and selected with geNorm 3.5 and StepOnePlus. Expression levels are indicated by fold change values and compared to the WT mice on the vehicle control. Details of the primers used are shown in Table 1.

### 4.10. Hepatic Neutral Lipid Quantification

Tissue Tek-embedded livers were cut with a cryostat CM3050S (Leica, Wetzlar, Germany) to obtain 14 µm sections which were mounted on SuperFrost Plus adhesion slides (Thermo Fisher Scientific, Waltham, MA, USA), air-dried overnight, and stored at room temperature until use. For hepatic neutral lipid staining, liver sections were fixed in 4% neutral buffered formalin, washed with tap water, and rinsed with 60% isopropanol. Hepatic lipids were stained with Oil Red O (Polysiences Inc., Warrington, FL, USA) for 15 min. Next, the liver sections were rinsed with 60% isopropanol, lightly stained with hematoxylin, and covered with a coverslip. Digital images of the sections were obtained using a Leica DMLB microscope (Leica Microsystems, Rijswijk, The Netherlands) equipped with software from the Leica Applications Suite (Leica Microsystems, Rijswijk, The Netherlands).

### 4.11. Statistical Analyses

All statistical analyses were performed using IBM SPSS Statistics 25. The Shapiro–Wilk normality test was used to test normal distribution. Unless stated otherwise, normally distributed data are presented as mean ± SD and analyzed using two-way ANOVA (with treatment and genotype as independent variables) and the Tukey post hoc test. Not-normally distributed data are presented as median (25th–75th percentile) and analyzed using the Mann–Whitney U test. The OLT and ORT discrimination index D2 and the fold change values of CCF-STTG1 cells (compared to the DMEM-F12 medium control) were analyzed using a one-sample T-test. Animals that did not reach the minimum of 4 s of exploration in T1 or T2 were excluded from further analyses. Extreme values were excluded using Dixon’s principles of exclusion of extreme values [56,57]. Significance are denoted as follows: * *p* ≤ 0.05, ** *p* ≤ 0.01, and *** *p* ≤ 0.001.

## Figures and Tables

**Table 1:** List of used primers and their corresponding forward (F) and reverse (R) nucleotide sequences.

Gene Symbol	Gene Name	Forward and Reverse Primer Sequence
**Mouse Organ Samples**		
*Abca1*	ATP-binding cassette, sub-family A, member 1	F: ACA TGA GTG CCA CTT TCC GA R: AGC AGG GGT TGT TGG CAT TA
*Abcg1*	ATP binding cassette, subfamily G, member 1	F: AAG GTC TCC AAT CTC GTG CC R: TCC ATG ACA AAG TCT GCT GGG
*Abcg5*	ATP binding cassette, subfamily G, member 5	F: CCT GCT GAG GCG AGT AAC AA R: GGA CGC GGA GAA GGT AGA AA
*Abcg8*	ATP binding cassette, subfamily G, member 8	F: ACA ACC TGT GGA TAG TGC CTG R: TTG AAT CTG CAT CAG CCC CG
*Actb*	Actin Beta	F: TTC TTG GGT ATG GAA TCC TGT GG R: GTC TTT ACG GAT GTC AAC GTC AC
*Apoe*	Apolipoprotein E	F: CAA GAA CTG ACG GCA CTG ATG R: TGT TCC TCC AGC TCC TTT TTG T
*App*	Amyloid beta (A4) precursor protein	F: GTC ATG ACT ATC CTC CTG GTG G R: GTG GAT ACC CCC TCC CCC AGC CTA GAC C
*B2m*	Beta-2-Microglobulin	F: CAT GGC TCG CTC GGT GAC C R: AAT GTG AGG CGG GTG GAA CTG
*Fasn*	Fatty Acid Synthase	F: CCC CTC TGT TAA TTG GCT CC R: TTG TGG AAG TGC AGG TTA GG
*Hprt1*	Hypoxanthine guanine phosphoribosyl transferase	F: CCT AAG ATG AGC GCA AGT TGA A R: CCA CAG GAC TAG AAC ACC TGC TAA
*Lipc*	Lipase C, hepatic type	F: ACG GGT GGT CGG TGG AT R: ATT CAC AGG TTG GGA CTG TCG
*Nr1h3 (Lxra)*	Nuclear receptor subfamily 1, group H, member 3 (Oxysterols receptor LXR-alpha)	F: AAC AGC TCC CTG GCT TCC TA R: CAG AAG CAT GAC CTC GAT TGC
*Plin2*	Perilipin 2	F: AGC CAA CGT CCG AGA TTG TT R: CTC CAG CCA TGG TAG TCG TC
*Scd1*	Stearoyl-Coenzyme A desaturase 1	F: GGC CTG TAC GGG ATC ATA CTG R: GGT CAT GTA GTA GAA AAT CCC GAA G
*Sdha*	Succinate dehydrogenase complex flavoprotein subunit A	F: CTT GAA TGA GGC TGA CTG TG R: ATC ACA TAA GCT GGT CCT GT
*Srebf1*	Sterol regulatory element-binding transcription factor 1	F: CAC ACA AAA GCA AAT CAC TGA AGG R: TCT CCA CCA CTT CGG GTT TC
**Human Caucasian astrocytoma cells (CCF-STTG1)**		
*ABCA1*	ATP-binding cassette, subfamily A, member 1	F: TCT CTG TTC GGC TGA GCT AC R: TGC AGA GGG CAT GGC TTT AT
*ABCG1*	ATP-binding cassette, subfamily G, member 1	F: GGT CGC TCC ATC ATT TGC AC R: GCA GAC TTT TCC CCG GTA CA
*APOE*	Apolipoprotein E	F: ACC CAG GAA CTG AGG GC R: CTC CTT GGA CAG CCG TG
*SDHA*	Succinate dehydrogenase complex flavoprotein subunit A	F: TGG GAA CAA GAG GGC ATC TG R: CCA CCA CTG CAT CAA ATT CAT G

## Data Availability

Data are available upon request.

## Supplementary Materials

The following are available online at https://www.mdpi.com/article/10.3390/md19040190/s1, Supplementary Figure S1: No effect of 24(S)-saringosterol on the expression of LXR target genes *Abca1, Abcg1, Apoe, Scd1*, and *Srebf1* in the hippocampus; Supplementary Figure S2: 24(S/R)-Saringosterol administration does not induce hepatic neutral lipid accumulation, hypertriglyceridemia, or hypercholesterolemia in C57BL6/J mice; Supplementary Table S1: Sterol concentrations in serum and tissues of C57BL6/J mice that received 24(S)-saringosterol via oral gavage (p.o.) and subcutaneous injection (s.c.); Supplementary Table S2: Randomization scheme for the object recognition task (ORT); Supplementary Table S3. Randomization scheme for the object location task (OLT).

## References

[1] Swerdlow R.H. (2007). Pathogenesis of Alzheimer’s disease. Clin. Interv. Aging.

[2] Cummings J., Aisen P.S., Dubois B., Frölich L., Jack C.R., Jones R.W., Morris J.C., Raskin J., Dowsett S.A., Scheltens P. (2016). Drug development in Alzheimer’s disease: The path to 2025. Alzheimers Res. Ther..

[3] Jansen D., Janssen C.I., Vanmierlo T., Dederen P.J., van Rooij D., Zinnhardt B., Nobelen C.L.M., Janssen A.-L., Hafkemeijer A., Mutsaers M.P.C. (2012). Cholesterol and synaptic compensatory mechanisms in Alzheimer’s disease mice brain during aging. J. Alzheimers Dis..

[4] Jones L., Holmans P.A., Hamshere M.L., Harold D., Moskvina V., Ivanov D., Pocklington A., Abraham R., Hollingworth P., Sims R. (2010). Genetic evidence implicates the immune system and cholesterol metabolism in the aetiology of Alzheimer’s disease. PLoS ONE.

[5] Kölsch H., Heun R., Jessen F., Popp J., Hentschel F., Maier W., Lütjohann D. (2010). Alterations of cholesterol precursor levels in Alzheimer’s disease. Biochim. Biophys. Acta.

[6] Mulder M. (2009). Sterols in the central nervous system. Curr. Opin. Clin. Nutr. Metab. Care.

[7] Loera-Valencia R., Goikolea J., Parrado-Fernandez C., Merino-Serrais P., Maioli S. (2019). Alterations in cholesterol metabolism as a risk factor for developing Alzheimer’s disease: Potential novel targets for treatment. J. Steroid Biochem. Mol. Biol..

[8] Vanmierlo T., Bloks V.W., Zee L.C.V.V.-V.D., Rutten K., Kerksiek A., Friedrichs S., Sijbrands E., Steinbusch H.W., Kuipers F., Lütjohann D. (2010). Alterations in Brain Cholesterol Metabolism in the APPSLxPS1mut mouse, a Model for Alzheimer’s Disease. J. Alzheimers Dis..

[9] Verghese P.B., Castellano J.M., Holtzman D.M. (2011). Apolipoprotein E in Alzheimer’s disease and other neurological disorders. Lancet Neurol..

[10] Kinney J.W., Bemiller S.M., Murtishaw A.S., Leisgang A.M., Salazar A.M., Lamb B.T. (2018). Inflammation as a central mechanism in Alzheimer’s disease. Alzheimers Dement..

[11] Zelcer N., Tontonoz P. (2006). Liver X receptors as integrators of metabolic and inflammatory signaling. J. Clin. Investig..

[12] Xu X., Xiao X., Yan Y., Zhang T. (2021). Activation of liver X receptors prevents emotional and cognitive dysfunction by suppressing microglial M1-polarization and restoring synaptic plasticity in the hippocampus of mice. Brain Behav. Immun..

[13] Jiang Q., Lee C.D., Mandrekar S., Wilkinson B., Cramer P., Zelcer N., Mann K., Lamb B., Willson T.M., Collins J.L. (2008). ApoE promotes the proteolytic degradation of Abeta. Neuron.

[14] Vanmierlo T., Rutten K., Dederen J., Bloks V.W., Zee L.C.V.V.-V.D., Kuipers F., Kiliaan A., Blokland A., Sijbrands E.J., Steinbusch H. (2011). Liver X receptor activation restores memory in aged AD mice without reducing amyloid. Neurobiol. Aging.

[15] Riddell D.R., Zhou H., Comery T.A., Kouranova E., Lo C.F., Warwick H.K., Ring R.H., Kirksey Y., Aschmies S., Xu J. (2007). The LXR agonist TO901317 selectively lowers hippocampal Abeta42 and improves memory in the Tg2576 mouse model of Alzheimer’s disease. Mol. Cell. Neurosci..

[16] Navas Guimaraes M.E., Lopez-Blanco R., Correa J., Fernandez-Villamarin M., Bistué M.B., Martino-Adami P., Morelli L., Kumar V., Wempe M.F., Cuello A.C. (2021). Liver X Receptor Activation with an Intranasal Polymer Therapeutic Prevents Cognitive Decline without Altering Lipid Levels. ACS Nano.

[17] Lefterov I., Bookout A., Wang Z., Staufenbiel M., Mangelsdorf D., Koldamova R. (2007). Expression profiling in APP23 mouse brain: Inhibition of Abeta amyloidosis and inflammation in response to LXR agonist treatment. Mol. Neurodegener..

[18] Grefhorst A., Elzinga B.M., Voshol P.J., Plo T., Kok T., Bloks V.W., van der Sluijs F.H., Havekes L.M., Romijn J.A., Verkade H.J. (2002). Stimulation of Lipogenesis by Pharmacological Activation of the Liver X Receptor Leads to Production of Large, Triglyceride-rich Very Low Density Lipoprotein Particles. J. Biol. Chem..

[19] Repa J.J., Liang G., Ou J., Bashmakov Y., Lobaccaro J.-M.A., Shimomura I., Shan B., Brown M.S., Goldstein J.L., Mangelsdorf D.J. (2000). Regulation of mouse sterol regulatory element-binding protein-1c gene (SREBP-1c) by oxysterol receptors, LXRalpha and LXRbeta. Genes Dev..

[20] Schultz J.R., Tu H., Luk A., Repa J.J., Medina J.C., Li L., Schwendner S., Wang S., Thoolen M., Mangelsdorf D.J. (2000). Role of LXRs in control of lipogenesis. Genes Dev..

[21] Bogie J., Hoeks C., Schepers M., Tiane A., Cuypers A., Leijten F., Chintapakorn Y., Suttiyut T., Pornpakakul S., Struik D. (2019). Dietary Sargassum fusiforme improves memory and reduces amyloid plaque load in an Alzheimer’s disease mouse model. Sci. Rep..

[22] Ngo D.-H., Vo T.-S., Wijesekara I., Kim S.-K. (2012). Biological activities and potential health benefits of bioactive peptides derived from marine organisms. Int. J. Biol. Macromol..

[23] Yende S.R., Harle U.N., Chaugule B.B. (2014). Therapeutic potential and health benefits of Sargassum species. Pharmacogn. Rev..

[24] Schepers M., Martens N., Tiane A., Vanbrabant K., Liu H.-B., Lütjohann D., Mulder M., Vanmierlo T. (2020). Edible seaweed-derived constituents: An undisclosed source of neuroprotective compounds. Neural Regen. Res..

[25] Wang X., Sun G., Feng T., Zhang J., Huang X., Wang T., Xie Z., Chu X., Yang J., Wang H. (2019). Sodium oligomannate therapeutically remodels gut microbiota and suppresses gut bacterial amino acids-shaped neuroinflammation to inhibit Alzheimer’s disease progression. Cell Res..

[26] Gao Y., Li C., Yin J., Shen J., Wang H., Wu Y., Jin H. (2012). Fucoidan, a sulfated polysaccharide from brown algae, improves cognitive impairment induced by infusion of Aβ peptide in rats. Environ. Toxicol. Pharmacol..

[27] Hu P., Li Z., Chen M., Sun Z., Ling Y., Jiang J., Huang C. (2016). Structural elucidation and protective role of a polysaccharide from Sargassum fusiforme on ameliorating learning and memory deficiencies in mice. Carbohydr. Polym..

[28] Jung H.A., Ali M.Y., Choi R.J., Jeong H.O., Chung H.Y., Choi J.S. (2016). Kinetics and molecular docking studies of fucosterol and fucoxanthin, BACE1 inhibitors from brown algae Undaria pinnatifida and Ecklonia stolonifera. Food Chem. Toxicol..

[29] Oh J.H., Choi J.S., Nam T.-J. (2018). Fucosterol from an Edible Brown Alga Ecklonia stolonifera Prevents Soluble Amyloid Beta-Induced Cognitive Dysfunction in Aging Rats. Mar. Drugs.

[30] Andrade P.B., Barbosa M., Matos R.P., Lopes G., Vinholes J., Mouga T., Valentão P. (2013). Valuable compounds in macroalgae extracts. Food Chem..

[31] Xiang S., Liu F., Lin J., Chen H., Huang C., Chen L., Zhou Y., Ye L., Zhang K., Jin J. (2017). Fucoxanthin Inhibits beta-Amyloid Assembly and Attenuates beta-Amyloid Oligomer-Induced Cognitive Impairments. J. Agric. Food Chem..

[32] Alghazwi M., Smid S., Musgrave I., Zhang W. (2019). In vitro studies of the neuroprotective activities of astaxanthin and fucoxanthin against amyloid beta (Abeta1-42) toxicity and aggregation. Neurochem. Int..

[33] Zhao X., Zhang S., An C., Zhang H., Sun Y., Li Y., Pu X. (2015). Neuroprotective effect of fucoxanthin on β-amyloid-induced cell death. J. Chin. Pharm. Sci..

[34] Jang E.J., Kim S.C., Lee J.H., Lee J.R., Kim I.K., Baek S.Y., Kim Y.W. (2018). Fucoxanthin, the constituent of Laminaria japonica, triggers AMPK-mediated cytoprotection and autophagy in hepatocytes under oxidative stress. BMC Complementary Altern. Med..

[35] Sangeetha R.K., Bhaskar N., Baskaran V. (2009). Comparative effects of beta-carotene and fucoxanthin on retinol deficiency induced oxidative stress in rats. Mol. Cell. Biochem..

[36] Chen Z., Liu J., Fu Z., Ye C., Zhang R., Song Y., Zhang Y., Li H., Ying H., Liu H. (2014). 24(S)-Saringosterol from edible marine seaweed Sargassum fusiforme is a novel selective LXRbeta agonist. J. Agric. Food Chem..

[37] Ye J.Y., Li L., Hao Q.M., Qin Y., Ma C.S. (2020). beta-Sitosterol treatment attenuates cognitive deficits and prevents amyloid plaque deposition in amyloid protein precursor/presenilin 1 mice. Korean J. Physiol. Pharmacol..

[38] Wang J., Wu F., Shi C. (2013). Substitution of membrane cholesterol with β-sitosterol promotes nonamyloidogenic cleavage of endogenous amyloid precursor protein. Neuroscience.

[39] Burg V.K., Grimm H.S., Rothhaar T.L., Grösgen S., Hundsdörfer B., Haupenthal V.J., Zimmer V.C., Mett J., Weingärtner O., Laufs U. (2013). Plant sterols the better cholesterol in Alzheimer’s disease? A mechanistical study. J. Neurosci..

[40] Koivisto H., Grimm M.O., Rothhaar T.L., Berkecz R., Lütjohann D., Giniatullina R., Takalo M., Miettinen P.O., Lahtinen H.-M., Giniatullin R. (2014). Special lipid-based diets alleviate cognitive deficits in the APPswe/PS1dE9 transgenic mouse model of Alzheimer’s disease independent of brain amyloid deposition. J. Nutr. Biochem..

[41] Kruit J.K., Plösch T., Havinga R., Boverhof R., Groot P.H., Groen A.K., Kuipers F. (2005). Increased fecal neutral sterol loss upon liver X receptor activation is independent of biliary sterol secretion in mice. Gastroenterology.

[42] Yu L., York J., von Bergmann K., Lutjohann D., Cohen J.C., Hobbs H.H. (2003). Stimulation of Cholesterol Excretion by the Liver X Receptor Agonist Requires ATP-binding Cassette Transporters G5 and G8. J. Biol. Chem..

[43] Ghisletti S., Huang W., Ogawa S., Pascual G., Lin M.-E., Willson T.M., Rosenfeld M.G., Glass C.K. (2007). Parallel SUMOylation-Dependent Pathways Mediate Gene- and Signal-Specific Transrepression by LXRs and PPARγ. Mol. Cell.

[44] Minett T., Cfas M., Classey J., Matthews F.E., Fahrenhold M., Taga M., Brayne C., Ince P.G., Nicoll J.A.R., Boche D. (2016). Microglial immunophenotype in dementia with Alzheimer’s pathology. J. Neuroinflamm..

[45] Simpson J.E., Ince P.G., Higham C.E., Gelsthorpe C.H., Fernando M.S., Matthews F., Forster G., O’Brien J.T., Barber R., Kalaria R.N. (2007). Microglial activation in white matter lesions and nonlesional white matter of ageing brains. Neuropathol. Appl. Neurobiol..

[46] Hopperton K.E., Mohammad D., Trépanier M.O., Giuliano V., Bazinet R.P. (2018). Markers of microglia in post-mortem brain samples from patients with Alzheimer’s disease: A systematic review. Mol. Psychiatry.

[47] Guo L., Ladu M.J., Van Eldik L.J. (2004). A Dual Role for Apolipoprotein E in Neuroinflammation: Anti- and Pro-Inflammatory Activity. J. Mol. Neurosci..

[48] Liu R., Hao D., Xu W., Li J., Li X., Shen D., Sheng K., Zhao L., Xu W., Gao Z. (2019). β-Sitosterol modulates macrophage polarization and attenuates rheumatoid inflammation in mice. Pharm. Biol..

[49] Yang E.-J., Ahn S., Ryu J., Choi M.-S., Choi S., Chong Y.H., Hyun J.-W., Chang M.-J., Kim H.-S. (2015). Phloroglucinol Attenuates the Cognitive Deficits of the 5XFAD Mouse Model of Alzheimer’s Disease. PLoS ONE.

[50] Philippou E., Constantinou M. (2014). The Influence of Glycemic Index on Cognitive Functioning: A Systematic Review of the Evidence. Adv. Nutr..

[51] Haskell-Ramsay C.F., Jackson P.A., Dodd F.L., Forster J.S., Bérubé J., Levinton C., Kennedy D.O. (2018). Acute Post-Prandial Cognitive Effects of Brown Seaweed Extract in Humans. Nutrients.

[52] Butterfield D.A., Halliwell B. (2019). Oxidative stress, dysfunctional glucose metabolism and Alzheimer disease. Nat. Rev. Neurosci..

[53] Lütjohann D., Brzezinka A., Barth E., Abramowski D., Staufenbiel M., von Bergmann K., Beyreuther K., Multhaup G., Bayer T.A. (2002). Profile of cholesterol-related sterols in aged amyloid precursor protein transgenic mouse brain. J. Lipid Res..

[54] Şık A., van Nieuwehuyzen P., Prickaerts J., Blokland A. (2003). Performance of different mouse strains in an object recognition task. Behav. Brain Res..

[55] Batenburg W.W., van den Heuvel M., van Esch J.H., van Veghel R., Garrelds I.M., Leijten F., Danser A.H. (2013). The (pro)renin receptor blocker handle region peptide upregulates endothelium-derived contractile factors in aliskiren-treated diabetic transgenic (mREN2)27 rats. J. Hypertens..

[56] Dixon W.J. (1951). Ratios Involving Extreme Values. Ann. Math. Stat..

[57] Dixon W.J. (1950). Analysis of Extreme Values. Ann. Math. Stat..

