# Supplementary material for: 24(S)-Saringosterol Prevents Cognitive Decline in a Mouse Model for Alzheimer’s Disease
Source: Mar Drugs. 2021 Mar 27;19(4):190. doi: 10.3390/md19040190 (PMC8065937; doi:10.3390/md19040190)
Supplement: Supplementary file 1 [file marinedrugs-19-00190-s001.pdf]

Supplemental figures and tables

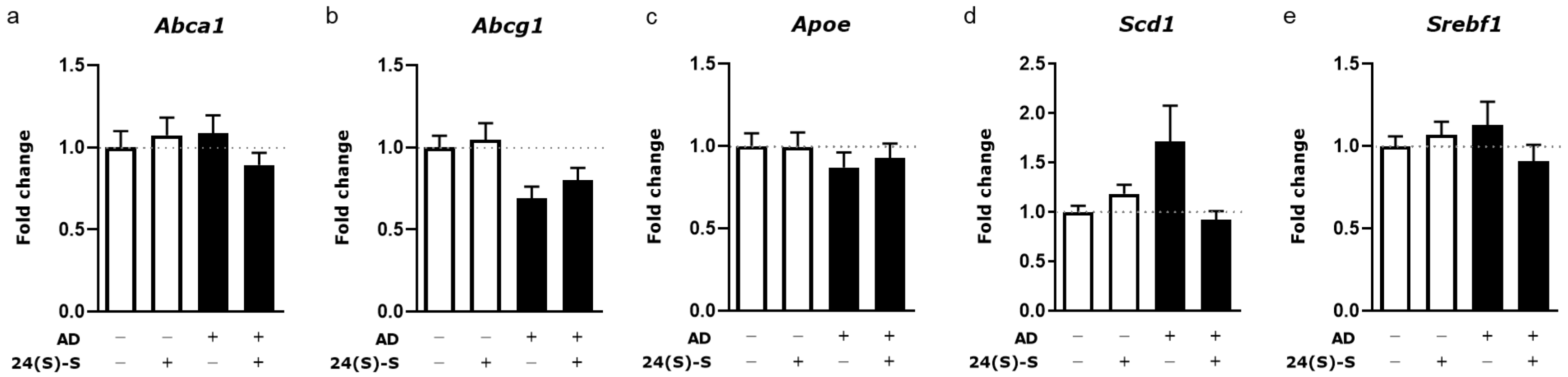

Supplementary Figure S1. No effect of 24(S)-saringosterol on the expression of LXR target genes *Abca1, Abcg1, Apoe, Scd1*, and *Srebf1* in the hippocampus. Gene expression of *Abca1* (a), *Abcg1* (b), *ApoE* (c), *Scd1*(d) and *Srebf1* (e) was analyzed in the brains of WT and APPswePS1ΔE9 mice treated with 24(S)-saringosterol or vehicle. Gene expression was normalized to the most stable reference genes (*Actb*, *B2m*, *Hprt1*, and *Sdha*) and expressed as fold change compared to WT mice treated with vehicle.

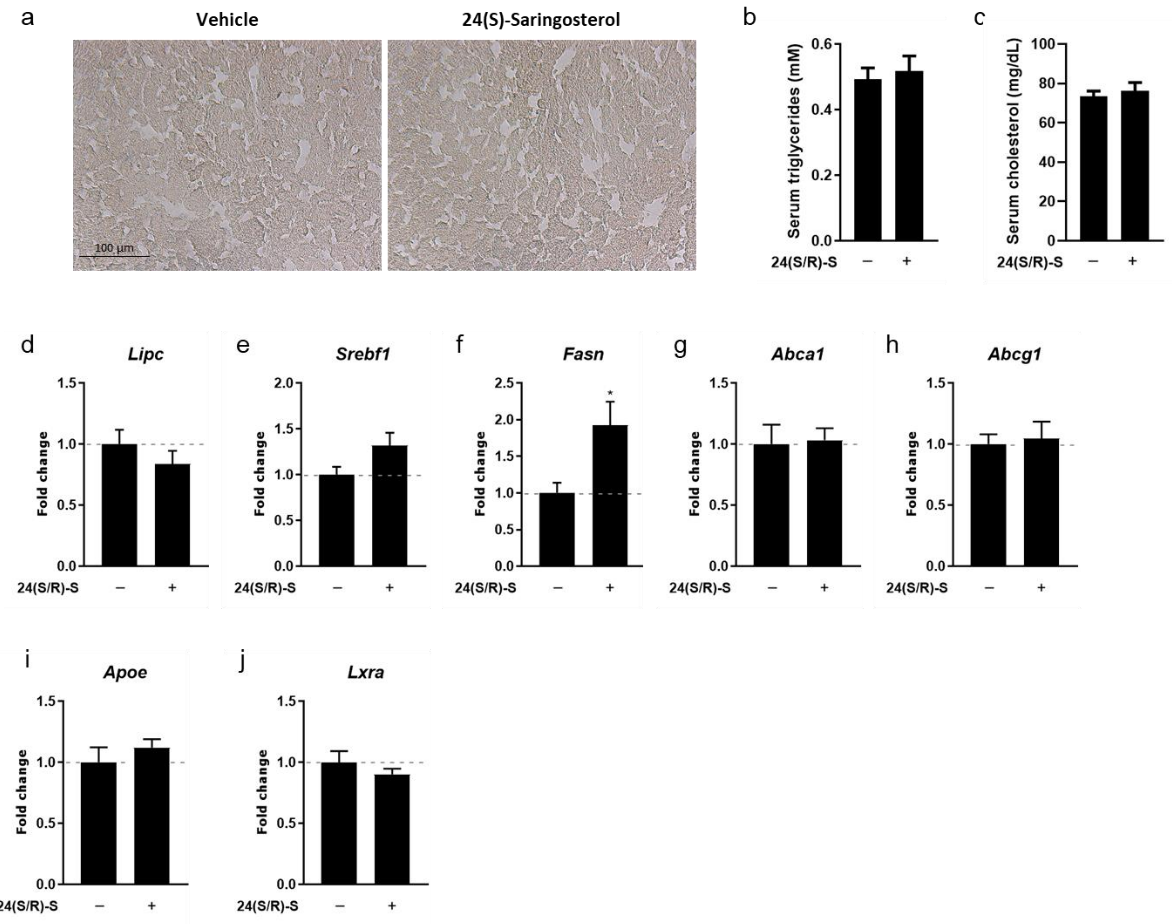

Supplementary Figure S2. 24(S/R)-Saringosterol administration does not induce hepatic neutral lipid accumulation, hypertriglyceridemia, or hypercholesterolemia in C57BL6/J mice. Representative Oil-Red-O-stained livers (**a**) and triglyceride (**b**) and cholesterol (**c**) concentrations in the serum of C57BL6/J mice are shown (n = 7 per treatment). The expression of the genes *Lipc* (d), *Srebf1* (e), *Fasn* (f), *Abca1* (g), *Abcg1* (h), *Apoe* (i), *Lxra* (j) in the livers of C57BL6/J mice is presented as fold changes (n ≥ 6 per treatment (*Abcg1:* vehicle-treated C57BL6/J mice: n = 4). The bars represent mean ± SEM.

Supplementary Table S1. Sterol concentrations in serum and tissues of C57BL6/J mice that received 24(S)-saringosterol via oral gavage (p.o.) and subcutaneous injection (s.c.)

		Cholestanol [mg/dL]	Lathosterol [µg/dL]	Campesterol [mg/dL]	Campestanol [µg/dL]	Stigmasterol [µg/dL]	Sitosterol [mg/dL]	Sitostanol [µg/dL]	Brassicasterol [µg/dL]	Lanosterol [µg/dL]	Desmosterol [µg/dL]	Dihydro-Lanosterol [µg/dL]	24OH-Cholesterol [ng/mL]	7aOH-Cholesterol [ng/mL]	27OH-Cholesterol [ng/mL]	Cholesterol(GC) [mg/dL]	Saringosterol [µg/dL]
Serum	p.o.	0.23	12.00	0.85	7.28	2.69	0.30	2.92	6.08	12.11	59.30	0.05	16.44	21.98	61.16	58.13	41.70
		0.28	10.68	1.23	9.97	2.91	0.41	3.58	6.27	11.82	50.64	0.06	16.84	19.19	73.15	59.90	48.09
		0.13	8.40	0.84	7.59	2.39	0.31	3.53	7.03	14.58	41.94	0.02	13.10	19.39	45.98	30.49	26.40
		0.21	11.48	0.96	8.82	3.32	0.31	3.32	6.29	11.28	56.00	0.04	19.25	27.53	60.10	50.01	58.57
		0.19	10.39	0.86	7.53	2.90	0.31	3.11	6.11	11.47	57.73	0.04	14.93	18.12	45.06	48.83	44.72
		0.35	15.10	1.49	11.59	2.56	0.45	4.08	6.53	11.84	52.99	0.06	12.77	27.84	54.38	72.61	25.99
	**Mean**	**0.23**	**11.34**	**1.04**	**8.80**	**2.79**	**0.35**	**3.42**	**6.38**	**12.18**	**53.10**	**0.04**	**15.56**	**22.34**	**56.64**	**53.33**	**40.91**
	**SD**	**0.08**	**2.22**	**0.27**	**1.70**	**0.33**	**0.07**	**0.41**	**0.35**	**1.21**	**6.30**	**0.01**	**2.46**	**4.33**	**10.56**	**14.08**	**12.74**
	s.c.	0.60	32.64	2.35	19.63	3.03	0.82	6.02	10.52	16.95	87.17	0.28	19.98	46.53	94.94	115.61	34.02
		0.40	23.02	2.14	17.56	2.88	0.76	5.36	9.67	14.19	65.36	0.13	15.05	30.63	76.79	79.04	25.74
		0.31	36.51	1.30	9.98	2.20	0.39	3.93	7.38	14.02	35.49	0.15	14.58	66.94	82.62	71.18	26.06
		0.55	14.96	2.22	17.30	2.33	0.66	4.75	9.38	12.74	47.70	0.14	15.23	39.45	63.82	83.17	26.23
		0.32	18.73	1.36	10.84	1.91	0.45	3.91	7.79	12.40	39.45	0.08	12.20	39.51	52.27	60.69	26.26
		0.54	14.46	1.99	15.04	1.96	0.56	4.62	8.05	11.46	42.00	0.06	17.93	31.79	95.08	91.24	29.48
	**Mean**	**0.45**	**23.39**	**1.89**	**15.06**	**2.39**	**0.61**	**4.77**	**8.80**	**13.63**	**52.86**	**0.14**	**15.83**	**42.47**	**77.59**	**83.49**	**27.97**
	**SD**	**0.13**	**9.28**	**0.45**	**3.89**	**0.47**	**0.17**	**0.82**	**1.24**	**1.92**	**19.80**	**0.08**	**2.73**	**13.32**	**17.11**	**18.88**	**3.27**

	Cholestanol [ng/mg]	Lathosterol [ng/mg]	Campesterol [ng/mg]	Campestanol [ng/mg]	Stigmasterol [ng/mg]	Sitosterol [ng/mg]	Sitostanol [ng/mg]	Brassicasterol [ng/mg]	Lanosterol [ng/mg]	Desmosterol [ng/mg]	Dihydro-Lanosterol [ng/mg]	24OH Cholesterol [ng/mg]	7aOH Cholesterol [ng/mg]	27OH Cholesterol [ng/mg]	s(GC) [µg/mg]	Saringosterol [ng/mg]

Cerebellum	p.o.	160.88	71.45	49.20	0.49	1.28	10.32	0.25	0.77	3.87	33.78	0.39	170.11		0.95	67.05	7.34
		155.05	74.81	57.95	0.58	1.48	12.27	0.24	0.69	4.28	35.20	0.43	160.43		0.95	66.89	7.81
		164.82	82.64	64.77	0.60	1.83	14.13	0.25	0.78	4.60	40.22	0.50	158.26		0.98	68.02	6.62
		174.99	90.03	55.38	0.55	1.85	12.38	0.25	0.67	5.07	35.88	0.53	174.21		1.18	73.36	6.91
		158.98	79.68	48.70	0.50	1.91	12.34	0.24	0.63	4.38	35.38	0.45	169.36		1.10	68.30	6.07
		176.47	67.67	88.42	0.85	1.35	18.75	0.27	1.12	3.91	30.36	0.42	145.82		0.87	66.31	3.84
	**Mean**	**165.20**	**77.71**	**60.74**	**0.60**	**1.61**	**13.36**	**0.25**	**0.77**	**4.35**	**35.14**	**0.45**	**163.03**		**1.00**	**68.32**	**6.43**
	**SD**	**8.76**	**8.11**	**14.81**	**0.13**	**0.28**	**2.90**	**0.01**	**0.18**	**0.45**	**3.20**	**0.05**	**10.40**		**0.11**	**2.58**	**1.40**
	s.c.	216.94	83.98	98.15	0.91	1.61	20.63	0.30	1.64	4.95	31.69	0.56	170.16		0.98	77.09	6.25
		188.52	80.10	87.89	0.80	1.29	18.27	0.29	1.21	5.14	32.97	0.52	153.30		0.99	70.81	4.77
		184.57	82.44	80.75	0.74	1.60	16.70	0.27	1.14	4.34	30.64	0.50	150.24		1.16	71.70	5.79
		193.97	63.33	101.91	0.95	1.64	19.57	0.30	1.28	3.81	23.26	0.44	163.42		1.08	67.22	8.68
		188.77	73.62	88.42	0.85	1.40	19.29	0.30	1.20	4.13	30.01	0.54	148.05		0.99	69.09	3.91
		188.50	79.95	99.48	0.84	1.67	20.56	0.28	1.30	5.22	33.80	0.66	149.70		1.06	69.88	3.67
	**Mean**	**193.55**	**77.24**	**92.77**	**0.85**	**1.53**	**19.17**	**0.29**	**1.30**	**4.60**	**30.40**	**0.54**	**155.81**		**1.04**	**70.96**	**5.51**
	**SD**	**11.85**	**7.68**	**8.30**	**0.08**	**0.15**	**1.49**	**0.01**	**0.18**	**0.59**	**3.77**	**0.07**	**8.93**		**0.07**	**3.37**	**1.85**
Liver	p.o.	3.74	3.21	86.50	0.96	0.58	21.54	0.36	0.89	1.98	6.63	0.07	0.18	1.02	2.37	6.11	8.95
		4.45	2.58	101.76	1.19	0.56	23.55	0.33	0.71	1.38	3.54	0.07	0.12	0.64	1.92	5.81	10.05
		4.12	2.26	106.64	1.21	0.61	26.17	0.39	0.99	1.75	4.81	0.05	0.14	0.75	1.64	6.20	10.01
		4.98	2.66	118.35	1.50	0.95	28.99	0.49	1.02	1.64	5.08	0.04	0.19	0.84	2.68	7.19	16.78
		4.08	2.07	94.29	1.11	0.61	23.44	0.34	0.77	1.30	3.62	0.05	0.15	0.57	2.06	6.26	11.97
		6.04	4.26	139.63	2.07	0.70	27.78	0.99	0.80	1.84	4.04	0.12	0.16	1.25	1.99	8.12	7.96
	**Mean**	**4.57**	**2.84**	**107.86**	**1.34**	**0.67**	**25.25**	**0.49**	**0.86**	**1.65**	**4.62**	**0.07**	**0.16**	**0.84**	**2.11**	**6.61**	**10.95**
	**SD**	**0.83**	**0.80**	**18.97**	**0.40**	**0.14**	**2.87**	**0.25**	**0.12**	**0.26**	**1.17**	**0.03**	**0.02**	**0.25**	**0.36**	**0.87**	**3.15**
	s.c.	7.51	7.65	193.23	2.34	0.58	50.77	1.01	1.57	3.84	5.07	0.33	0.14	1.19	2.08	9.06	5.82
		7.06	6.24	194.09	2.70	0.69	50.09	1.09	1.67	3.62	5.30	0.20	0.16	1.06	2.19	8.25	6.90
		5.99	11.85	136.88	1.89	0.58	32.00	0.95	1.09	3.31	3.23	0.50	0.13	1.06	2.10	8.56	5.84
		8.22	4.14	203.80	2.51	0.52	44.26	0.87	1.41	2.54	3.89	0.20	0.13	0.91	2.02	8.16	6.49

		6.73	6.79	197.21	2.71	0.68	45.77	1.17	1.38	3.24	4.78	0.29	0.12	1.23	2.20	8.54	7.81
		8.69	3.04	213.70	2.74	0.54	40.33	1.09	1.24	1.72	2.96	0.08	0.15	0.86	2.04	8.97	8.07
	**Mean**	**7.37**	**6.62**	**189.82**	**2.48**	**0.60**	**43.87**	**1.03**	**1.39**	**3.05**	**4.20**	**0.26**	**0.14**	**1.05**	**2.10**	**8.59**	**6.82**
	**SD**	**0.99**	**3.08**	**27.03**	**0.33**	**0.07**	**6.98**	**0.11**	**0.21**	**0.78**	**0.99**	**0.14**	**0.01**	**0.15**	**0.07**	**0.37**	**0.96**
**Intestine**	**p.o.**	61.59	20.15	89.39	8.10	3.88	45.48	6.79	0.70	3.19	162.98	0.04	0.25	0.43	0.36	11.63	15.71
		62.12	12.33	97.26	1.92	0.76	24.94	0.61	0.78	3.00	134.57	0.03	0.27	0.37	0.53	10.59	19.36
		66.51	11.11	87.53	1.72	0.71	25.83	0.63	0.89	3.55	216.21	0.03	0.22	0.48	0.38	12.96	13.67
		72.00	13.69	79.58	1.49	0.78	22.29	0.57	0.77	3.64	222.01	0.04	0.31	0.41	0.47	13.60	18.85
		60.65	20.43	96.57	14.67	7.14	67.45	14.67	0.82	4.14	221.17	0.05	0.27	0.39	0.39	12.07	37.83
		66.62	8.06	118.49	1.73	0.41	29.98	0.79	1.12	3.05	214.32	0.02	0.20	0.42	0.61	12.54	10.66
	**Mean**	**64.92**	**14.29**	**94.80**	**4.94**	**2.28**	**36.00**	**4.01**	**0.85**	**3.43**	**195.21**	**0.04**	**0.25**	**0.42**	**0.46**	**12.23**	**19.35**
	**SD**	**4.31**	**5.00**	**13.30**	**5.41**	**2.71**	**17.48**	**5.77**	**0.15**	**0.44**	**37.19**	**0.01**	**0.04**	**0.04**	**0.10**	**1.06**	**9.62**
	**s.c.**	84.49	41.77	119.21	1.99	0.44	30.85	0.74	1.02	5.12	193.13	0.07	0.18	0.37	0.38	14.52	5.78
		65.31	10.15	106.16	1.60	0.36	28.53	0.64	1.16	3.78	194.91	0.04	0.28	0.40	1.09	11.73	7.00
		79.71	26.89	98.99	2.38	0.61	26.36	1.41	1.03	3.71	260.88	0.04	0.28	0.47	0.78	13.42	6.90
		82.55	18.06	120.39	3.47	0.93	32.24	2.47	0.99	3.15	98.50	0.03	0.22	0.34	0.62	11.43	5.94
		75.52	16.58	112.80	11.36	4.08	53.96	14.96	1.04	2.99	117.89	0.03	0.22	0.35	0.50	12.76	6.94
		67.47	9.82	118.14	1.93	0.32	27.76	0.64	0.91	2.44	69.59	0.02	0.23	0.33	1.04	10.48	6.47
	**Mean**	**75.84**	**20.54**	**112.62**	**3.79**	**1.12**	**33.28**	**3.48**	**1.02**	**3.53**	**155.82**	**0.04**	**0.23**	**0.38**	**0.73**	**12.39**	**6.50**
	**SD**	**7.95**	**12.13**	**8.51**	**3.77**	**1.47**	**10.35**	**5.67**	**0.08**	**0.92**	**72.27**	**0.02**	**0.04**	**0.05**	**0.29**	**1.47**	**0.53**
**White fat**	**p.o.**	2.02	1.73	8.72	0.21	0.15	3.52	0.19	0.41	1.64	3.40	0.01	0.38	0.12	0.87	0.63	13.11
		2.48	0.78	12.63	0.28	0.16	4.64	0.20	0.60	1.94	3.47	0.01	0.43	0.13	0.91	0.66	15.52
		2.57	1.16	13.83	0.21	0.16	4.36	0.13	0.34	1.43	5.18	0.01	0.37	0.11	0.88	0.69	14.82
		2.94	0.56	14.20	0.28	0.20	4.77	0.19	0.45	1.59	8.37	0.01	0.65	0.11	1.13	0.87	18.93
		1.98	0.89	8.84	0.26	0.17	3.83	0.18	0.52	1.85	5.28	0.01	0.35	0.13	0.59	0.60	11.34
		2.43	0.91	15.07	0.30	0.18	5.04	0.22	0.55	1.51	2.44	0.01	0.38	0.12	1.01	0.75	10.71
	**Mean**	**2.40**	**1.00**	**12.22**	**0.26**	**0.17**	**4.36**	**0.18**	**0.48**	**1.66**	**4.69**	**0.01**	**0.43**	**0.12**	**0.90**	**0.70**	**14.07**
	**SD**	**0.36**	**0.41**	**2.77**	**0.04**	**0.02**	**0.58**	**0.03**	**0.10**	**0.20**	**2.11**	**0.00**	**0.11**	**0.01**	**0.18**	**0.10**	**3.03**

	s.c.	3.61	0.70	19.90	0.23	0.11	5.46	0.14	0.32	1.20	2.50	0.01	0.35	0.13	1.21	0.87	5.79
		12.38	2.53	35.16	0.59	0.28	8.83	0.30	0.58	1.98	503.92	0.01	0.37	0.25	1.15	1.82	5.57
		4.00	3.31	21.45	0.43	0.20	6.18	0.23	0.55	1.62	3.54	0.01	0.63	0.17	2.84	1.23	8.75
		3.81	1.56	20.91	0.26	0.13	4.95	0.16	0.39	1.65	2.89	0.01	0.48	0.11	1.86	0.83	4.98
		2.96	1.56	16.48	0.25	0.12	4.82	0.15	0.35	1.32	3.75	0.01	0.34	0.11	1.13	0.74	5.62
		3.82	0.94	23.52	0.24	0.14	5.88	0.16	0.37	1.27	1.71	0.01	0.51	0.11	1.96	0.85	5.48
	**Mean**	**5.10**	**1.77**	**22.90**	**0.33**	**0.16**	**6.02**	**0.19**	**0.43**	**1.51**	**86.38**	**0.01**	**0.45**	**0.15**	**1.69**	**1.06**	**6.03**
	**SD**	**3.59**	**0.99**	**6.43**	**0.15**	**0.07**	**1.47**	**0.06**	**0.11**	**0.30**	**204.55**	**0.00**	**0.11**	**0.06**	**0.67**	**0.41**	**1.36**
**Testis**	p.o.	219.45	58.76	129.68	4.84	1.47	35.76	0.83	1.48	6.89	385.15	0.03	1.50	2.88	1.34	11.90	9.16
		205.81	54.50	170.19	6.15	1.47	41.38	0.83	1.62	6.89	277.94	0.03	1.51	3.70	1.44	10.53	7.92
		149.24	53.97	160.85	5.36	1.36	41.27	0.82	1.60	6.78	310.48	0.04	1.75	3.37	1.60	8.05	6.65
		185.19	58.67	160.30	5.04	1.60	38.65	0.93	1.57	7.72	283.98	0.04	1.96	2.46	2.12	10.72	9.96
		118.79	55.35	137.42	4.71	1.19	34.93	0.73	1.41	6.95	277.29	0.03	1.95	2.78	1.42	6.05	6.86
		112.51	44.34	166.02	5.19	0.75	36.84	1.08	1.81	6.00	267.58	0.02	1.54	3.12	1.36	7.41	3.97
	**Mean**	**165.17**	**54.26**	**154.08**	**5.22**	**1.31**	**38.14**	**0.87**	**1.58**	**6.87**	**300.40**	**0.03**	**1.70**	**3.05**	**1.54**	**9.11**	**7.42**
	**SD**	**45.11**	**5.28**	**16.49**	**0.52**	**0.31**	**2.77**	**0.12**	**0.14**	**0.55**	**43.98**	**0.01**	**0.22**	**0.44**	**0.29**	**2.27**	**2.12**
	s.c.	205.06	52.05	202.83	6.28	0.71	44.52	1.25	2.37	6.79	311.88	0.03	2.47	3.38	1.74	13.79	3.08
		230.11	80.76	306.16	2.60	1.34	68.51	2.08	3.28	11.83	485.58	0.04	3.47	6.65	2.78	14.47	4.81
		86.55	61.56	123.49	4.29	0.58	29.41	0.96	1.48	5.90	262.31	0.03	1.50	1.71	1.37	5.06	3.49
		151.44	38.76	233.10	9.98	0.81	45.01	1.52	2.84	5.58	237.39	0.03	1.83	4.01	1.39	6.45	3.40
		102.53	47.41	164.31	5.43	0.75	40.54	1.24	1.90	6.46	235.72	0.02	1.91	2.43	1.56	5.92	3.65
		63.52	42.89	185.20	6.41	0.66	40.17	1.30	2.07	4.88	242.85	0.02	2.09	4.21	1.58	3.53	3.01
	**Mean**	**139.87**	**53.91**	**202.52**	**5.83**	**0.81**	**44.69**	**1.39**	**2.32**	**6.91**	**295.95**	**0.03**	**2.21**	**3.73**	**1.74**	**8.21**	**3.57**
	**SD**	**67.22**	**15.34**	**62.74**	**2.48**	**0.27**	**12.95**	**0.38**	**0.65**	**2.51**	**97.18**	**0.01**	**0.70**	**1.72**	**0.53**	**4.70**	**0.65**
**Adrenal**	p.o.	760.45	41.45	922.34	34.22	3.71	98.00	3.75	11.98	22.98	38.14	0.88	0.92	25.77	0.92	33.95	103.62
		549.25	18.73	737.20	28.32	2.43	68.74	2.91	9.01	12.30	19.04	0.40	0.75	23.17	1.16	22.99	88.13
		574.98	28.81	780.41	28.11	2.49	82.56	3.50	10.18	15.77	27.60	0.48	0.77	13.73	1.29	25.74	81.95
		385.77	22.25	445.55	16.72	2.16	52.80	2.40	5.59	10.77	24.78	0.37	0.96	6.91	1.20	16.34	80.62

		297.15	16.79	335.98	12.17	1.66	44.44	1.70	4.17	7.85	23.55	0.30	0.64	5.67	0.81	13.88	59.98
		1001.66	38.61	1492.06	64.03	2.81	125.11	6.06	16.40	27.63	43.22	1.21	0.66	20.29	0.87	44.26	61.25
	**Mean**	**594.88**	**27.77**	**785.59**	**30.60**	**2.54**	**78.61**	**3.38**	**9.55**	**16.22**	**29.39**	**0.61**	**0.78**	**15.92**	**1.04**	**26.19**	**79.26**
	**SD**	**256.02**	**10.38**	**409.37**	**18.30**	**0.69**	**29.95**	**1.51**	**4.43**	**7.63**	**9.31**	**0.36**	**0.13**	**8.48**	**0.20**	**11.37**	**16.60**
	**s.c.**	351.06	20.58	536.04	21.29	1.12	60.31	2.46	7.27	16.45	13.77	0.82	0.51	8.97	1.04	15.04	30.15
		299.26	28.32	652.74	18.85	1.39	85.92	2.75	8.33	11.08	15.78	0.51	0.56	5.14	1.64	16.59	32.45
		336.31	36.47	538.72	13.96	1.12	63.36	2.20	6.89	8.92	17.51	0.36	0.66	4.87	2.29	20.35	35.54
		852.81	43.41	1543.73	51.68	2.34	151.19	5.81	21.72	21.36	12.42	0.96	0.78	13.43	1.35	37.29	47.81
		852.68	46.83	1337.02	42.56	3.20	154.22	5.79	18.87	20.18	22.10	0.81	0.69	31.07	0.99	41.37	54.59
		715.53	41.90	1433.97	43.75	1.98	134.96	5.26	17.50	17.68	14.98	0.86	0.78	10.53	1.69	35.35	30.39
	**Mean**	**567.94**	**36.25**	**1007.04**	**32.01**	**1.86**	**108.33**	**4.05**	**13.43**	**15.95**	**16.09**	**0.72**	**0.66**	**12.33**	**1.50**	**27.67**	**38.49**
	**SD**	**267.16**	**10.04**	**478.72**	**15.81**	**0.82**	**43.55**	**1.75**	**6.66**	**4.97**	**3.42**	**0.23**	**0.11**	**9.74**	**0.49**	**11.62**	**10.26**
**Feces**	**p.o.**	27.69	18.86	341.16	287.78	118.56	1055.30	355.93	5.64	18.43	11.08	0.12	1.84	0.41	0.32	0.98	2.99
		54.40	17.89	434.05	390.47	154.21	1267.06	519.47	6.62	25.13	9.99	0.21	1.38	0.34	0.33	1.07	3.33
		34.41	19.17	418.10	337.75	139.79	1196.92	417.81	6.50	21.13	10.53	0.18	1.30	0.49	0.21	1.38	12.16
		33.97	16.35	458.83	373.28	167.16	1341.09	471.46	7.75	19.03	9.77	0.14	1.77	0.43	0.45	1.30	1953.99
		39.02	8.50	397.61	365.45	142.96	1211.57	460.55	7.13	25.54	12.66	0.22	2.15	0.33	0.65	0.92	692.09
		41.72	21.37	299.12	288.53	119.12	947.15	364.52	5.72	15.75	11.92	0.10	0.98	0.34	0.16	0.64	21.16
	**Mean**	**38.54**	**17.02**	**391.48**	**340.54**	**140.30**	**1169.85**	**431.62**	**6.56**	**20.84**	**10.99**	**0.16**	**1.57**	**0.39**	**0.35**	**1.05**	**447.62**
	**SD**	**9.14**	**4.49**	**60.26**	**44.00**	**19.20**	**144.19**	**64.13**	**0.81**	**3.89**	**1.13**	**0.05**	**0.43**	**0.07**	**0.18**	**0.27**	**786.83**
	**s.c.**	53.11	153.32	859.00	823.42	297.61	2930.19	1428.12	10.17	32.16	19.56	0.23	1.11	0.46	0.23	0.95	3.61
		48.54	51.82	899.01	981.54	362.00	3260.89	1714.52	13.72	42.04	13.61	0.36	2.66	0.56	0.51	1.04	11.61
		40.77	44.55	748.62	775.93	292.66	2526.71	1309.59	6.39	27.08	8.08	0.14	1.17	1.73	0.23	0.78	2.37
		50.03	25.08	830.19	847.14	300.34	2985.25	1484.37	11.02	33.18	11.59	0.21	2.04	0.30	0.44	0.82	4.42
		49.94	32.71	521.72	595.34	196.73	1700.38	1025.38	6.62	21.50	7.85	0.14	0.96	0.23	0.21	0.71	2.88
		98.26	57.06	837.06	822.19	276.51	2583.85	1416.47	14.54	25.18	12.33	0.23	2.27	0.40	0.57	0.89	4.61
	**Mean**	**56.78**	**60.76**	**782.60**	**807.59**	**287.64**	**2664.55**	**1396.41**	**10.41**	**30.19**	**12.17**	**0.22**	**1.70**	**0.61**	**0.36**	**0.86**	**4.92**
	**SD**	**20.74**	**46.87**	**136.99**	**125.17**	**53.30**	**544.83**	**226.07**	**3.43**	**7.26**	**4.30**	**0.08**	**0.71**	**0.56**	**0.16**	**0.12**	**3.39**

Supplementary Table S2. Randomization scheme for the object recognition task (ORT)

	1		2		3		4		5		6		7		8	
Mouse	T1	T2	T1	T2	T1	T2	T1	T2	T1	T2	T1	T2	T1	T2	T1	T2
1/13/25/37	**11**	**12**	**22**	**32**	**33**	**34**	**44**	**14**	**33**	**43**	**44**	**41**	**11**	**21**	**22**	**23**
2/14/26/38	**22**	**23**	**33**	**43**	**44**	**41**	**11**	**21**	**44**	**14**	**11**	**12**	**22**	**32**	**33**	**34**
3/15/27/39	**33**	**34**	**44**	**14**	**11**	**12**	**22**	**32**	**11**	**21**	**22**	**23**	**33**	**43**	**44**	**41**
4/16/28/40	**44**	**41**	**11**	**21**	**22**	**23**	**33**	**43**	**22**	**32**	**33**	**34**	**44**	**14**	**11**	**12**
5/17/29/41	**11**	**12**	**22**	**32**	**33**	**34**	**44**	**14**	**33**	**43**	**44**	**41**	**11**	**21**	**22**	**23**
6/18/30/42	**22**	**23**	**33**	**43**	**44**	**41**	**11**	**21**	**44**	**14**	**11**	**12**	**22**	**32**	**33**	**34**
7/19/31/43	**33**	**43**	**44**	**41**	**11**	**21**	**22**	**23**	**11**	**12**	**22**	**32**	**33**	**34**	**44**	**14**
8/20/32/44	**44**	**14**	**11**	**12**	**22**	**32**	**33**	**34**	**22**	**23**	**33**	**43**	**44**	**41**	**11**	**21**
9/21/33/45	**11**	**21**	**22**	**23**	**33**	**43**	**44**	**41**	**33**	**34**	**44**	**14**	**11**	**12**	**22**	**32**
10/22/34/46	**22**	**32**	**33**	**34**	**44**	**14**	**11**	**12**	**44**	**41**	**11**	**21**	**22**	**23**	**33**	**43**
11/23/35/47	**33**	**43**	**44**	**41**	**11**	**21**	**22**	**23**	**11**	**12**	**22**	**32**	**33**	**34**	**44**	**14**
12/24/36/48	**44**	**14**	**11**	**12**	**22**	**32**	**33**	**34**	**22**	**23**	**33**	**43**	**44**	**41**	**11**	**21**

(grey) = Right object is novel

(white) = Left object is novel

During the first trial (T1), the animal was exposed to two similar objects (object 1, 2, 3, or 4) for 4 minutes after which it was placed back in its home cage. After a 1h inter-trial interval, the second trial (T2) was performed during which the animals was exposed for 4 minutes to a familiar object (from T1) and a novel object. The exploration time of both objects in T1 and T2 were recorded and used to calculate the discrimination index (D2) (([exploration time for novel object] – [exploration time for familiar object] / [total exploration time in T2]) as a measure of object memory.

Supplementary Table S3. Randomization scheme for the object location task (OLT)

	1		2		3		4		5		6		7		8	
Mouse	T1	T2	T1	T2	T1	T2	T1	T2	T1	T2	T1	T2	T1	T2	T1	T2
1/13/25/37	**11**	**1F**[1]	**22**	**B2**	**33**	**3B**	**44**	**F4**	**33**	**B3**	**44**	**4F**	**11**	**F1**	**22**	**2B**
2/14/26/38	**22**	**2F**	**33**	**B3**	**44**	**4B**	**11**	**F1**	**44**	**B4**	**11**	**1F**	**22**	**F2**	**33**	**3B**
3/15/27/39	**33**	**3F**	**44**	**B4**	**11**	**1B**	**22**	**F2**	**11**	**B1**	**22**	**2F**	**33**	**F3**	**44**	**4B**
4/16/28/40	**44**	**4B**[2]	**11**	**F1**	**22**	**2F**	**33**	**B3**	**22**	**F2**	**33**	**3B**	**44**	**B4**	**11**	**1F**
5/17/29/41	**11**	**1B**	**22**	**F2**	**33**	**3F**	**44**	**B4**	**33**	**F3**	**44**	**4B**	**11**	**B1**	**22**	**2F**
6/18/30/42	**22**	**2B**	**33**	**F3**	**44**	**4F**	**11**	**B1**	**44**	**F4**	**11**	**1B**	**22**	**B2**	**33**	**3F**
7/19/31/43	**33**	**F3**	**44**	**4B**	**11**	**B1**	**22**	**2F**	**11**	**1B**	**22**	**F2**	**33**	**3F**	**44**	**B4**
8/20/32/44	**44**	**F4**	**11**	**1B**	**22**	**B2**	**33**	**3F**	**22**	**2B**	**33**	**F3**	**44**	**4F**	**11**	**B1**
9/21/33/45	**11**	**F1**	**22**	**2B**	**33**	**B3**	**44**	**4F**	**33**	**3B**	**44**	**F4**	**11**	**1F**	**22**	**B2**
10/22/34/46	**22**	**B2**	**33**	**3F**	**44**	**F4**	**11**	**1B**	**44**	**4F**	**11**	**B1**	**22**	**2B**	**33**	**F3**
11/23/35/47	**33**	**B3**	**44**	**4F**	**11**	**F1**	**22**	**2B**	**11**	**1F**	**22**	**B2**	**33**	**3B**	**44**	**F4**
12/24/36/48	**44**	**B4**	**11**	**1F**	**22**	**F2**	**33**	**3B**	**22**	**2F**	**33**	**B3**	**44**	**4B**	**11**	**F1**

(grey box) = Right object is displaced

(white box) = Left object is displaced

[1] F: Front, [2] B: Back

During the first trial (T1), the animal was for 4 minutes exposed to two similar objects (object 1, 2, 3, or 4) placed symmetrically in the arena center after which it was placed back in its home cage. After a 4h inter-trial interval, the second trial (T2) was performed during which the animals was exposed for 4 minutes to the two objects from T1 of which one was placed forward (F) or placed back (B). The exploration time of both objects in T1 and T2 were recorded and used to

calculate the discrimination index (D2) (([exploration time for novel object] – [exploration time for familiar object] / [total exploration time in T2]) as a measure of spatial memory.
